# Potential Applications of Chitosan and Chitosan-Based Materials in Farm Animal Reproductive Management: Direct and Indirect Implications

**DOI:** 10.3390/polym18050616

**Published:** 2026-02-28

**Authors:** Eman M. Hassanein, Ottó Szenci

**Affiliations:** 1 Department of Obstetrics and Farm Animal Medicine Clinic, University of Veterinary Medicine, István u. 2, H-1078 Budapest, Hungary; em.mostafa@alexu.edu.eg; 2 Animal and Fish Production Department, Faculty of Agriculture (El-Shatby), Alexandria University, Alexandria 21545, Egypt

**Keywords:** chitosan, drug delivery system, dietary supplement, estrus synchronization, semen preservation, uterine health, metritis/endometritis, gut health, reproductive performance, livestock

## Abstract

Chitosan (CH) is a natural biopolymer obtained from the deacetylation process of chitin found in the exoskeleton of crustaceans. Recently, CH has been used as a multifunctional molecule in farm animal health, production, and reproduction. CH has an exceptional chemical structure and physicochemical properties that confer valuable properties, such as biocompatibility, biodegradability, antimicrobial and antioxidant activities, immune modulation, mucoadhesion, and controlled release capabilities. These properties enable CH to be formulated in various forms, including raw CH, chitosan oligosaccharides (COSs), microparticles, nanoparticles (NPs), solutions, gels, and films, thereby expanding its applicability for improving fertility and enhancing reproductive performance in farm animals. Several reports have described various applications of CH and CH-based materials in animal reproduction, including dietary supplementation, sperm preservation, in vitro embryo production (IVEP), treatment of uterine infections such as metritis/endometritis, and integration into synchronization protocols as a hormone delivery system. Therefore, this review outlines the potential applications of CH and CH-based materials to improve reproductive performance in farm animals through both direct and indirect mechanisms.

## 1. Introduction

Efficient reproductive performance is a critical component for sustainable and profitable farm animal production systems. However, the livestock industry encounters several reproductive challenges associated with intensive management practices, environmental and metabolic stressors, infertility, infectious diseases, and continuous demand for genetic improvement through assisted reproductive technologies (ARTs) [1,2]. These challenges have motivated ongoing research to identify effective, safe, and multifunctional biomaterials that can preserve and support reproductive processes across different physiological stages, from gametogenesis and gamete preservation to pregnancy maintenance and offspring performance [3].

In recent years, natural polymers have attracted increasing attention in the livestock industry due to their unique characteristics, including biocompatibility, biodegradability, and the ability to interact safely with biological systems [4]. Polysaccharides are naturally occurring biopolymers widely distributed in plants, animals, and microorganisms, and play structural roles [5]. They are complex carbohydrates composed of long chains of monosaccharide units bonded by glycosidic bonds [6]. Recently, polysaccharides have emerged as promising candidates in biological and biomedical fields due to their various bioactive functions, including antioxidant, antimicrobial, antiviral, anti-inflammatory, antitumor, hypoglycemic, and immunomodulatory properties [6,7]. Among these, chitin, a major structural component of crustacean and insect exoskeletons and the second-most-abundant polysaccharide in nature after cellulose, has attracted significant attention [8,9,10]. Chitin is a linear polymer composed mainly of β-(1→4)-linked N-acetyl-D-glucosamine units [11]. Its highly crystalline structure and extensive intra- and intermolecular hydrogen bonding make chitin insoluble in water and most organic solvents, limiting its processing and biological applicability despite its exceptional functional properties [11,12].

In this context, chitosan (CH) has emerged as a promising alternative because of its biocompatibility, biodegradability, low toxicity, and various biological activities. CH is a natural, readily available, and economical polysaccharide biopolymer composed of repeating β-(1→4)-linked D-glucosamine and N-acetyl-D-glucosamine units. It is produced through the deacetylation of chitin [8,9,10]. CH differs from chitin mainly due to the presence of free amino groups at the C-2 position of the glucosamine units, which confer a cationic character under acidic and slightly neutral conditions [10,13]. This cationic nature of CH enables a wide range of electrostatic interactions, contributing to its diverse functions and properties. As a result, CH exhibits broad-spectrum antimicrobial activity, principally due to its electrostatic binding to anionic components of microbial cell membranes, thereby disrupting them [14,15]. Additionally, CH exhibits mucoadhesive properties, which are useful for prolonged retention and stability in mucosal tissues [16]. It also displays antioxidant and anti-inflammatory properties, allowing it to reduce oxidative stress (OS) by scavenging free radicals [14,17]. Moreover, CH can be formulated into various physical forms, including powder, hydrogels, films, microparticles or microspheres, and nanoparticles (NPs) [18,19]. Each formulation exhibits distinct properties that can be customized for specific applications. Collectively, all of these properties have positioned CH and CH-based materials as promising functional biomaterials for a wide range of reproductive applications in farm animals [4,19,20,21,22].

While several reviews have summarized the potential applications of CH in veterinary medicine, most have focused mainly on its antimicrobial, wound-healing, therapeutic, or drug delivery properties in general animal health [23]. In farm animals, recent reviews have highlighted its role as a feed additive and growth-promoting agent [4,24], as well as its impact on digestibility, microbiota performance [24], metabolic responses, intestinal morphology, and nutrition [25]. Other reviews have also focused solely on using CH as a nanocarrier for drug delivery [26,27,28,29]. To our knowledge, no comprehensive review has particularly explained the direct and indirect impacts of CH and CH-based materials in farm animal reproduction. Therefore, this review primarily aims to: (1) summarize the physicochemical properties and biological activities of CH relevant to reproductive applications of farm animal species; (2) represent the direct effects of CH and CH-based materials on several reproductive management practices such as sperm preservation, in vitro embryo production, uterine health and hormonal synchronization; and (3) show the indirect reproductive benefits potentially mediated through enhancements in gut health, immune modulation and stress reduction.

## 2. Literature Search Strategy

Various databases, including PubMed, Web of Science, Scopus, and Google Scholar, were used to perform the literature search. The research was focused on studies published between 2000 and 2025. The search incorporated key terms, including “chitosan”, “chitosan oligosaccharides”, “chitosan nanoparticles”, “chitosan microparticles”, “farm animals”, “reproduction”, “fertility”, “sperm preservation”, “uterine health”, “metritis”, “endometritis”, “oocyte maturation”, “embryo production”, “drug delivery system”, and “estrus synchronization”, and relevant farm animal species such as “cattle”, “sheep”, “goat”, “swine”, “buffalo”, and “rabbit”. Additionally, subsequent searches were conducted to identify studies on rumen microbiota, gut health, immune modulation, and stress responses in farm animals, with a focus on the potential reproductive implications of CH. Furthermore, relevant publications reporting reproductive or physiologically related outcomes were included in this review. Manual screening of reference lists was also conducted to recognize further relevant studies. This search included peer-reviewed articles and early-access online publications (in press). Preprints were included only when relevant. After eliminating duplicates and assessing the relevant studies on the reproductive application of CH in farm animals, a total of 185 publications were included.

## 3. Chemical Structure and Physical Forms of Chitosan

CH is a natural, cationic, linear polysaccharide obtained from the chitin molecule via a partial deacetylation process. Structurally, CH consists of repeating β-(1→4)-linked D-glucosamine and N-acetyl-D-glucosamine units [8,9,10]. The degree of deacetylation (DD) indicates the proportion of free amino groups at the C-2 position of the D-glucosamine units along the polymer chain. In contrast, the molecular weight (MW) reflects the length of the polymer chain. Based on MW, CH is classified into low-MW (<100,000 Da), medium-MW (between 100,000 and 1,000,000 Da), and high-MW CH (>1,000,000 Da) [30]. Both DD and MW are critical determinants of the functional behavior of CH, as they influence solubility, charge density, viscosity, degradation rate, and biological activities. These characteristics emphasize the suitability of CH as a promising polymer for a wide range of biological applications [11,31,32].

Furthermore, depending on DD, MW, and processing conditions, CH can be fabricated into various physical forms, including raw powders, films, fibers, sponges, beads, membranes, scaffolds, microspheres, and NPs [32,33]. In animal production applications, CH is commonly utilized as raw powder, hydrogel, microspheres, or NPs, with the selected form determined by the administration route, the proposed application, and the target tissue [19].

Raw CH with a high MW contains long polymer chains that exhibit several properties, including mucoadhesion and an antimicrobial barrier effect, due to their high viscosity and low solubility. Its high viscosity makes it ideal for gel, film, and fiber formation, especially for drug delivery, wound healing, tissue repair, and proliferation. It is recognized as a safe, biocompatible, and biodegradable agent. However, its poor solubility and limited cellular penetration restrict its application [14,32]. CH hydrogels have been applied in reproductive management, particularly for intrauterine applications, drug delivery, and as antimicrobial barriers. Their high water retention capacity and compatibility with soft tissue make them appropriate for intrauterine and mucosal environment applications in farm animals [34,35].

In contrast, CH with a low MW has shorter polymer chains, lower viscosity, higher solubility, and improved bioavailability. These features enhance interactions with biological tissues, improving cell penetration, antioxidant, and antimicrobial effects [36]. Although it is safe due to natural biocompatibility, its activity depends on concentration, as high or excessive concentrations may adversely impact cell membranes or the local environment [36]. As a result, in animal nutrition, CH with a low MW at an optimal concentration is frequently incorporated as a feed supplement due to its digestibility and its ability to interact with the gut microbiota [24,37,38,39,40,41].

In addition, chitosan oligosaccharides (COSs), the hydrolyzed form of CH, have the lowest MW (<10,000 Da) formed by depolymerization and are commonly used in animal feeding strategies due to their rapid absorption and biological activity at low inclusion levels [19,42,43]. These properties are mainly attributed to their lower DD and MW, which confer higher water solubility, lower viscosity, and enhanced permeability across biological barriers compared to conventional CH [44,45]. COSs exhibit improved biological activities, such as antioxidant, anti-inflammatory, and metabolic modulation effects, because their short chains facilitate interactions with biological tissues, broadening their application range. Their safety is typically high due to their good biocompatibility [25,44].

CH-NPs are manufactured particles produced by ionic gelation methods. NPs have the highest efficacy due to their large surface-area-to-volume ratio, resulting in enhanced antimicrobial activity and superior bioavailability for drug delivery. In the context of ART, CH-NPs are widely used for their ability to encapsulate various biomolecules, including hormones, antioxidants, antimicrobial agents, and micronutrients [26,27,46]. This allows targeted, controlled release and enhanced bioavailability of the encapsulated agents [26,47]. While they are generally safe, their efficacy depends heavily on formulation, dose, timing, and disease status.

## 4. Biological Activities Supporting Farm Animal Reproduction

**Figure 1:**
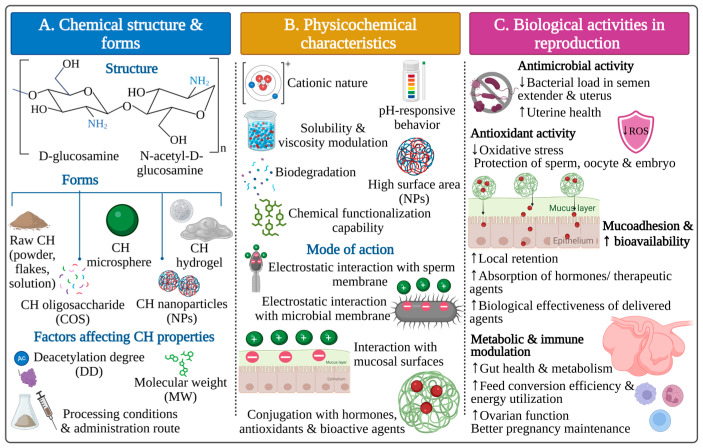
Illustration showing (**panel A**) the chemical structure, several forms, and factors affecting properties of chitosan (CH) and chitosan-based materials; (**panel B**) their physicochemical characteristics; and (**panel C**) the biological activities of CH and CH-based materials relevant to livestock reproduction. ↑: Increase, ↓: Decrease.

CH and CH-based materials exhibit various biological activities that support their utilization in the livestock industry, especially in animal reproduction (Figure 1). CH is a non-toxic, biocompatible, and biodegradable polymer that can be enzymatically degraded in biological environments into nontoxic monomers such as glucosamine, facilitating safe clearance and minimizing inflammatory responses [48].

### 4.1. Antimicrobial Activity

CH exhibits broad-spectrum antimicrobial activity, which is particularly valuable in the livestock industry, where microbial contamination can adversely impact several aspects, including sperm preservation quality, uterine health, and overall reproductive efficiency. This antimicrobial activity is primarily attributed to the cationic nature of CH, resulting from protonated amino groups, which enable strong electrostatic interactions with negatively charged microbial cell membranes [15,49]. These interactions lead to disruption of membrane integrity and fluidity, ultimately causing cell lysis [50,51]. In addition to membrane disruption, CH can inhibit microbial growth by interfering with mRNA and protein synthesis, resulting in disruption to transcription and translation processes. It may also form an extracellular barrier that limits and blocks nutrient and gas exchange, further suppressing microbial activity and proliferation [15,51,52]. The specific mechanism of action varies depending on the type of bacteria. In Gram-negative bacteria, electrostatic interactions between amino groups of CH (positively charged) and lipopolysaccharides (LPSs) in the outer membrane (negatively charged) lead to bacterial membrane destabilization and leakage of intracellular components [53,54]. In contrast, Gram-positive bacteria are affected by interactions between CH and teichoic acids in peptidoglycan on the bacterial surface, resulting in impaired cell wall barrier function [15,48,54].

Moreover, CH can display antifungal activity against specific fungal species by penetrating fungal hyphae and inhibiting the fundamental enzymes critical for growth and development [15,51]. These antimicrobial properties are essential for reducing bacterial and fungal contamination during semen preservation [51], boosting postpartum uterine health, and preventing microbial infections, thereby increasing reproductive efficiency [55,56]. Furthermore, by modulating gut microbiota and limiting pathogen load in the gastrointestinal tract, CH may indirectly support productive and reproductive performance [24].

The antimicrobial activity of CH is influenced by its MW. CH with a high MW acts at the extracellular level by altering membrane permeability, thereby limiting its ability to penetrate microbial cell membranes. Conversely, CH with a low MW can show both extracellular and intracellular antibacterial effects, including interference with RNA and protein synthesis as well as mitochondrial function [57].

### 4.2. Antioxidant Activity

Additionally, CH and CH-based materials exhibit notable antioxidant properties, acting as free radical scavengers and metal chelators [58]. Both mechanisms act synergistically to protect cells from OS by scavenging and neutralizing free radicals (hydroxyl, superoxide, and diphenyl picrylhydrazyl (DPPH)) and chelating metal ions, including iron (Fe^2+^) and copper (Cu^2+^), thereby inhibiting their participation in Fenton reactions and the formation of highly reactive hydroxyl radicals [48].

OS is a significant cause of sperm damage during cryopreservation. Changes in mitochondrial membrane fluidity and sperm membrane phospholipid composition promote excessive reactive oxygen species (ROS) production, surpassing the capacity of endogenous antioxidant defenses [59,60,61]. This imbalance initiates oxidative and nitrosative chain reactions, resulting in lipid peroxidation, mitochondrial dysfunction, DNA fragmentation, and damage to vital biomolecules, including carbohydrates, lipids, proteins, and nucleic acids, ultimately compromising sperm integrity and fertilization capacity [59,60,61]. OS also represents a considerable limitation to gametogenesis, oocyte competence, embryo development, and ovarian activity in farm animals [62].

By eliminating oxidative damage, CH contributes to improved cellular integrity and reproductive outcomes, particularly under heat stress or an intensive production system. Several studies have reported that the addition of CH or CH-based materials reduces lipid peroxidation and enhances antioxidant enzyme activity, such as superoxide dismutase (SOD), glutathione peroxidase (GPx), and catalase (CAT), while reducing levels of fatty acids and malondialdehyde (MDA) [63,64]. The antioxidant capacity of CH is also affected by DD and MW, with lower-MW and higher-DD CH exhibiting superior antioxidant activity due to a greater ability to disrupt oxidative chain reactions [65].

### 4.3. Mucoadhesive Properties

Furthermore, the mucoadhesive properties of CH and CH-based materials improve their retention on mucosal surfaces, such as uterine, vaginal, and gastrointestinal epithelia. This property is mainly dependent on the electrostatic interactions between cationic CH polymer and anionic components of mucin and mucosal membranes [66]. Enhanced mucoadhesion improves the local efficacy and bioavailability of CH and its delivered bioactive compounds, such as hormones and therapeutic agents. Additionally, CH has the ability to transiently modulate epithelial tight junctions, facilitating paracellular transport and further enhancing absorption and biological effectiveness [67]. The mucoadhesive capacity is influenced by both MW and DD, with a higher MW and DD being associated with stronger mucoadhesion, while excessive crosslinking tends to decrease this property [48]. CH-based hydrogels and mucoadhesive formulations are particularly relevant for intrauterine or intravaginal applications where prolonged residence times and localized drug action are required, such as in postpartum uterine therapy [34,35,68].

### 4.4. Drug Delivery

Regarding drug delivery, CH can be formulated into NPs or conjugated with other biomaterials, making it an effective carrier for drugs, hormones, antioxidants, vaccines, and therapeutic agents. Its ability to protect biomolecules, modulate release kinetics, and target specific tissue highlights its value as a drug delivery system [69]. Many reproductive drugs and hormones suffer from limited stability, rapid degradation, and low bioavailability. Using CH-based delivery systems offer a promising approach to protect these bioactive molecules from degradation, extend their bioavailability, provide sustained release to target organs, and facilitate their transport across biological barriers [27]. This approach can enhance cellular uptake of bioactive components, allowing for lower doses than conventional administration and benefiting substances such as hormones, peptides, and drugs [27,69,70,71].

Among CH-based delivery systems, NPs are the most widely used due to their small size, large surface area, and ability to encapsulate both hydrophilic and hydrophobic compounds. The physical characteristics of these carriers, including shape, size, and surface charge, play a critical role in determining their interaction with cells and tissues [67,70].

Through ionic gelation or ionic crosslinking, CH can form stable complexes with encapsulated biomolecules under specific conditions, enabling controlled and prolonged release. Additionally, CH can be combined with anionic polymers such as hyaluronic acid or alginate for extended release patterns [72,73]. Therefore, CH-based drug delivery systems are extensively applied in reproductive management of farm animals, including female reproductive applications such as ovulation induction [74,75,76,77], estrus synchronization [78,79,80,81], resumption of ovarian activity postpartum [82,83], and preventive uterine health management [34,68]. In male reproduction, CH-based systems have been used to deliver hormones that enhance spermatogenesis and fertility [84] and to deliver antioxidants during semen preservation [85,86,87,88,89].

## 5. Application of Chitosan and Chitosan-Based Materials in Reproduction

### 5.1. Semen Preservation

Cryopreservation is a common procedure designed to maintain cellular viability at lower temperatures for extended periods by suspending metabolic and enzymatic activity. In particular, sperm cryopreservation plays an essential role in the conservation of genetic resources in livestock and endangered species, enabling the global distribution of genetically superior germplasm without the need to transport live animals [90,91]. Moreover, semen cryopreservation is a cornerstone of ARTs, such as artificial insemination (AI), in vitro fertilization (IVF), and intracytoplasmic sperm injection (ICSI) [92].

Despite these advantages, both liquid semen storage and cryopreservation expose sperm to several stressors, including OS, microbial contamination, membrane destabilization, and structural and functional damage [93]. These stressors can compromise sperm quality and subsequent fertility outcomes. To mitigate these challenges, antibiotics and antioxidants have been traditionally incorporated into semen extenders. However, the extensive use of antibiotics has raised serious concerns regarding antimicrobial resistance, increasing the risk of disease transmission, disrupting the microbiome population of the female reproductive tract, and decreasing reproductive efficiency, highlighting the need for sustainable alternatives [94].

**Figure 2:**
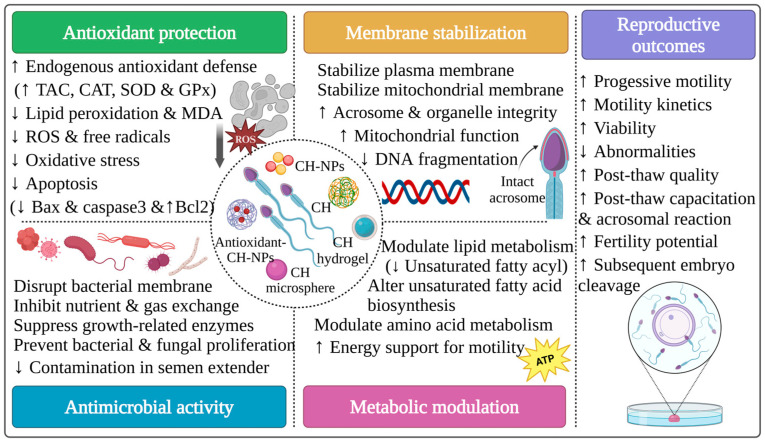
Diagram of the multifunctional roles and mechanisms of chitosan (CH) and chitosan-based materials in semen preservation, including antioxidant protection, membrane stabilization, antimicrobial activity, and metabolic modulation, and their impact on reproductive outcomes. ↑: Increase, ↓: Decrease.

In this context, CH and CH-based materials have emerged as promising multifunctional alternatives due to their antimicrobial, antifungal, antioxidant, membrane-stabilizing, and bioactive delivery features (Figure 2) [23]. Several studies have investigated different CH formulations in sperm preservation. However, due to variability in experimental objectives and conditions, concentrations used, and species differences, detailed study-specific mechanistic explanations are summarized in Table 1.

#### 5.1.1. Antimicrobial Activity

A recent study has demonstrated that raw CH exhibits natural antimicrobial activity and can effectively replace conventional antibiotics in semen extenders during storage. The inclusion of raw CH in a rabbit semen extender during refrigeration at 16 °C for 72 h markedly reduced bacterial load, especially *Enterococcus faecalis*, compared to media supplemented with penicillin and streptomycin, while maintaining sperm viability, membrane integrity, and overall semen quality [51]. Notably, the fertility rate following AI with CH-supplemented semen was comparable to that of those animals inseminated using synthetic antibiotic-supplemented extenders, confirming its practical applicability under commercial conditions [51]. The antimicrobial activity of CH is attributed to its cationic nature, which facilitates interaction with negatively charged bacterial membranes, leading to disruption of cell membrane integrity, interference with enzymatic activity, inhibition of nutrient and gas exchange, and prevention of microbial proliferation [95,96]. Furthermore, CH may exhibit antifungal effects by penetrating fungal hyphae and suppressing growth-related enzymatic pathways [15].

**Table 1:** Applications of chitosan (CH) and chitosan-based materials in semen preservation of farm animals.

CH Form	Species	Preservation Method	Extender	Optimal Concentration/Outcomes	Proposed Mechanism of CH
Raw CH [51,97]	Rabbit (bucks)	Liquid preservation (16 °C for 72 h)	TCG	0.05% (*w*/*v*) CH in semen extender: Exhibits similar sperm motility & viability ↓ Bacterial (Enterococcus Faecalis) load compared to conventional antibiotics	Antimicrobial, antifungal & antioxidant agent Disrupts bacterial membrane fluidity Inhibits fungal growth & developmental enzymes ↓ Lipid peroxidation & protects from OS
	Goat (bucks)	Liquid preservation (4 °C for 5 d)	Tris-based	0.2 mg/mL CH in semen extender: ↑ Sperm viability & antioxidant capacity ↑ Plasma membrane integrity ↑ Motility parameters	Antioxidant & antimicrobial agent Scavenges free radicals Supports the endogenous antioxidant system: (↑ TAC-CAT & ↓ ROS-MDA) Alters seminal plasma lipid metabolites
CH-NPs [64,98]	Sheep (rams)	Conventional slow freezing	Tris-based	15 µg/mL CH-NPs during cryopreservation: ↑ Post-thaw progressive motility, viability, acrosome & membrane integrity ↑ Antioxidant defense (TAC & SOD) ↓ Abnormalities Maintain ultrastructure integrity	Antioxidant, anti-inflammatory & biodegradable agent Scavenges ROS & free radicals ↑ Antioxidant enzyme activity (TAC & SOD) ↓ Lipid peroxidation, OS & MDA Stabilizes membrane & organelles Prevents DNA toxicity
	Cattle (dairy bulls)	Conventional slow freezing	Tris-based	20 µg/mL CH-NPs in capacitation media (TALP) with heparin: ↑ Post-thaw capacitation Hyperactivate motility & acrosome reaction after 60 min ↑ In vitro fertilization & embryo cleavage	Positively charged CH-NPs Interact with the negatively charged plasma membrane Modulate post-thaw membrane properties ↑ Capacitation changes (cholesterol efflux & ↑ membrane fluidity) Stabilize the membrane surface microenvironment
CH–dextran sulphate NPs [75]	Rabbit (bucks)	Liquid preservation (4 °C)	TCG	0.05–0.1% CH-NPs complex in extender: Maintain motility, viability & membrane functionality ↑ Sperm quality & acrosome integrity	Positive charged, non-toxic & biocompatible CH-NPs: Interact with the sperm plasma membrane ↑ Acrosome integrity
CH–hyaluronic acid (GnHA) (or) CH–hydrogel (GnHG) [99]	Swine (boars)	Conventional slow freezing	LEY	0.25 or 1.0 mg/mL GnHA in cryopreservation: ↑ Post-thaw motility & ↓ lipid peroxidation 0.5 mg/mL GnHG in cryopreservation: ↑ Motility parameters, activity & integrity ↓ MDA level and lipid peroxidation	Antioxidant & bioactive polymer ↓ ROS-induced lipid peroxidation (↓ MDA) Preserves membrane integrity & enhances motility Mitigates OS & cryo-damage GnHA supports membrane hydration & viscosity GnHG offers a matrix to stabilize sperm cells
Microencapsulated CH (or) microencapsulated CH + rrβ-NGF [93,94]	Rabbit (bucks)	Liquid preservation (RT for 30 min)	Incubation medium	0.5–1 µg/mL microsphere of CH or CH-rrβ-NGF in incubation media for 30 min: No adverse impact on viability & motility ↑ Capacitation & acrosomal reaction	CH protects rrβ-NGF from enzyme degradation rrβ-NGF acts via specific receptors (TrKA/p75) Promotes maturation, capacitation & acrosome reaction
Selenium–CH-NPs (nanozyme) [85]	Buffalo (bulls)	Conventional slow freezing	Tris-based	1–2 µg/mL nanozyme during preservation ↑ Post-thaw motility & viability ↓ OS Maintains ultrastructure integrity & mitochondrial function	Mimics natural enzymes (GPx/SOD) to detoxify ROS Activates Nrf2 for regulating endogenous antioxidants (GPx4, SOD & HO-1) Stabilizes membranes through cationic reaction ↓ Lipid peroxidation & mitochondrial damage
Rosmarinic acid–CH-NPs [86]	Buffalo (bulls)	Conventional slow freezing	Tris-based	100 µg/mL rosmarinic acid–CH-NPs during cryopreservation: ↑ Post-thaw motility, viability, acrosome & ultrastructure integrity ↑ Antioxidant defense (CAT & GPx) ↓ Abnormalities & apoptosis	Scavenge ROS & free radicals ↓ Lipid peroxidation & OS ↓ Apoptosis (↓ Bax/Caspase-3 & ↑ Bcl-2) NPs act as antimicrobial agents Modulate membrane fluidity & integrity
Silymarin–CH-NPs [87]	Buffalo (bulls)	Conventional slow freezing	Tris-based	100 µg/mL silymarin–CH-NPs during cryopreservation: ↑ Post-thaw sperm quality, motility, viability & in vivo fertility ↑ Oxidative status (TAC, SOD & GPx) ↓ Abnormalities & apoptosis Preserve ultrastructure integrity	Silymarin–CH-NPs act as antioxidant & antiapoptotic agents: Scavenge ROS & RNS ↓ Lipid peroxidation (↓ MDA) ↓ OS & support modulation of enzymes (↑ TAC, SOD & GPx) ↓ Apoptosis & necrosis (↓ Bax/Caspase-3 & ↑ Bcl-2) Enhance integrity, motility & fertility
Green tea extract–CH-NPs [88]	Goat (bucks)	Conventional slow freezing	Skim milk-based	1 µg/mL green tea extract–CH-NPs during cryopreservation: ↑ Post-thaw sperm motility, viability & integrity ↓ Post-thaw abnormalities	Green tea extract–CH-NPs acts as antioxidant agents: Scavenge ROS Act with the membrane to improve membrane stability ↓ Lipid peroxidation, oxidative- & cryo-damage ↑ Post-thaw semen quality
Epigallocatechin-3-gallate–CH-NPs [89]	Goat (bucks)	Conventional slow freezing	Skim milk-based	1.5–2 µg/mL epigallocatechin-3-gallate–CH-NPs during freezing: ↑ Post-thaw semen quality & integrity ↑ CAT & DPPH radical scavenging ↓ OS & MDA	Epigallocatechin-3-gallate–CH-NPs exhibit antioxidant protection: Chelate iron ions, inhibiting ROS generation Scavenge ROS & DPPH radicals ↑ Cellular uptake (↑ surface area of NPs) ↑ CAT activity & reduce H_2_O_2_ ↓ Lipid peroxidation & DNA fragmentation Preserve membrane and acrosome integrity

↑: Increase, ↓: Decrease, Bax: Bcl-2-associated X protein (pro-apoptotic), Bcl-2: B-cell lymphoma 2 (anti-apoptotic), Caspase-3: Cysteine-aspartic acid protease-3 (pro-apoptotic), CAT: Catalase, CH: Chitosan, CH-NPs: Chitosan nanoparticles, DNA: Deoxyribonucleic acid, DPPH: Diphenyl picrylhydrazyl, GPx: Glutathione peroxidase, H_2_O_2_: Hydrogen peroxide, HO-1: Heme oxygenase-1, LEY: Lactose–egg yolk extender, MDA: Malondialdehyde, NPs: Nanoparticles, OS: Oxidative stress, RNS: Reactive nitrogen species, ROS: Reactive oxygen species, rrβ-NGF: Recombinant rabbit β-nerve growth factor, RT: Room temperature, SOD: Superoxide dismutase, TAC: Total antioxidant capacity, TALP: Tyrod’s albumin lactate pyruvate, TCG: Tris–citric acid–glucose extender, TrkA: Tropomyosin receptor kinase A.

#### 5.1.2. Metabolic Effects

CH improves seminal plasma metabolism and sperm resilience during refrigeration [97]. In buck semen stored at 4 °C, supplementation with CH significantly improved sperm viability, motility parameters, structural integrity, and antioxidant status [97]. Notably, only one study suggested that CH could modulate lipid and amino acid metabolism in seminal plasma by decreasing unsaturated fatty acyls and oxidized lipids, while altering unsaturated fatty acid biosynthesis. Additionally, CH could increase the abundance of oligopeptides associated with sperm viability and integrity, thereby preserving membrane function and extending refrigerated semen preservation by up to 5 days [97].

#### 5.1.3. Antioxidant Protection

OS remains one of the significant causes of sperm damage during cryopreservation. Several studies have reported that CH and CH-based formulations exhibit effective antioxidant activity and markedly eliminate cryo-induced injuries across multiple species. In rams, the inclusion of CH-NPs in a frozen extender significantly improved post-thaw motility, viability, acrosome and membrane integrity, and antioxidant status, without negatively affecting DNA integrity, mainly by ROS scavenging mechanisms [64]. Comparable protective impacts have been observed in other species. For instance, an advanced nanozyme-based system (e.g., selenium-conjugated CH-NPs) further boosted post-thaw sperm quality and cryosurvival in buffaloes by mimicking endogenous antioxidant enzymes (SOD and GPx), activating redox-regulatory pathways, stabilizing mitochondrial membrane potential, and maintaining sperm ultrastructure [85].

The antioxidant efficacy of CH is also enhanced when it functions as a nanocarrier for antioxidants and bioactive compounds. Encapsulation of antioxidant molecules, including rosmarinic acid [86], silymarin [87], green tea extract [88], and epiallocatechin-3-gallate [89] within CH-NPs markedly improved their solubility, stability, bioavailability, and cellular uptake. These nano-formulations substantially reduced ROS, MDA, nitric oxide (NO), and apoptotic biomarkers, while increasing endogenous antioxidant enzyme activity and upregulating anti-apoptotic gene expression, ultimately resulting in improved sperm quality and fertilization potential [86,87,88,89].

#### 5.1.4. Plasma Membrane Stabilization

In addition to oxidative protection, CH contributes to semen preservation by directly interacting with the sperm plasma membrane. As a naturally cationic polymer, CH binds to anionic components of the plasma membrane, forming a stabilizing layer that maintains membrane fluidity and preserves acrosomal and structural integrity during cryopreservation [97]. Studies utilizing various CH-based formulations, including CH-NPs conjugated with sodium tripolyphosphate (TPP) [55,90] or dextran sulphate [66], CH hydrogels, and CH–hyaluronic acid complexes [91], in preservation media have reported enriched membrane hydration, increased viscosity, and enhanced cellular protection [64,75,98,99]. Moreover, these electrostatic interactions may facilitate cholesterol efflux, enhance membrane fluidity, and activate capacitation-associated signaling pathways. Supplementation of bovine sperm capacitation media with CH-NPs improved hyperactivated motility, acrosome reaction, and in vitro fertilization rates [98]. Similarly, microencapsulation of recombinant β-nerve growth factor (rβ-NGF) in CH microspheres, when added to the incubation media of rabbit sperm, maintained protein bioactivity, activated capacitation and the acrosome reaction without compromising sperm viability, and prevented premature acrosomal reaction induced by free nerve growth factor (NGF) [100,101]. These findings emphasize the potential of CH and CH-based materials to regulate sperm functional activation during AI and IVF protocols precisely.

Collectively, the literature demonstrates that CH and CH-based materials play multifunctional roles in semen preservation by improving sperm quality, maintaining structural and functional integrity, supporting controlled capacitation, and enhancing reproductive performance. In addition, their application offers a sustainable approach to reducing reliance on antibiotics and synthetic additives in ART applications.

Although the majority of studies report improvements in sperm functional, structural, and quality parameters, it is important to note that the current evidence is based on experimental in vitro studies. Also, relatively fewer studies have directly evaluated fertility outcomes. Therefore, while the available evidence supports the beneficial effects of CH and CH-based materials on sperm quality, evidence on actual fertility rates is promising but limited. Moreover, variability across studies regarding several factors, including CH forms, concentration used, and animal species, contributes to heterogeneity in reported outcomes.

### 5.2. Applications of Chitosan During In Vitro Embryo Production Stages

In vitro embryo production (IVEP) is a crucial ART in farm animal reproduction and consists of a sequential series of stages, including in vitro maturation (IVM), fertilization (IVF), and embryo culture (IVC). This approach is widely applied for the preservation of genetic resources, fertility management, genetic improvement programs, and embryo transfer programs [102,103]. Despite continuous improvements in maturation and culture systems, embryos produced in vitro often exhibit lower cleavage rates, developmental competence, and quality than their in vivo counterparts [94,95]. These differences are primarily attributed to suboptimal culture conditions, including OS, altered energy metabolism, and the absence of the optimal maternal reproductive tract environment [104]. Therefore, supplementation of maturation, fertilization, and embryo culture media with bioactive components has been widely used to maximize oocyte maturation, fertilization efficiency, and embryo development by mimicking optimal physiological conditions and reducing induced stress [103,105]. In this context, the inherent properties of CH and CH-based materials, including biocompatibility, biodegradability, antioxidant activity, and diverse physicochemical structures, make them promising supplements for boosting maturation and culture systems and protecting gametes and early embryos from oxidative and metabolic stress (Table 2).

#### 5.2.1. CH-NP Inclusion in the Maturation Stage

During the IVM stage, supplementation with CH-NPs (10 mg/mL) has been shown to provide superior protection and enhance oocyte quality and subsequent embryonic development, especially under stress conditions. In immature bovine oocytes exposed to linoleic acid, CH-NPs significantly alleviated the inhibitory effects of linoleic acid on nuclear maturation and cumulus cell expansion, restored blastocyst development, and reduced DNA damage in cumulus cells [106]. Similarly, porcine oocytes supplemented with 25 µg/mL CH-NPs during IVM stages exhibited elevated intracellular reduced glutathione (GSH) levels, reduced ROS accumulation, improved mitochondrial function, and increased maturation, cleavage, and blastocyst rates [107]. These findings indicate that CH-NPs help preserve redox balance and support cytoplasmic maturation processes critical for subsequent embryonic development.

#### 5.2.2. CH-NPs as Nanocarriers During the Maturation Stage

Moreover, CH-NPs have been utilized as nanocarriers for antioxidant delivery during the IVM stage. For instance, melatonin-loaded CH-NPs significantly improved the nuclear maturation rate of buffalo oocytes compared with free melatonin, along with upregulating genes associated with oocyte competence (growth differentiation factor 9 (GDF9) and bone morphogenetic protein 15 (BMP15)), antioxidant defense (Superoxide dismutase 1 (SOD1)), and cell survival (B-cell lymphoma 2 (Bcl-2)), while downregulating pro-apoptotic gene expression (Bcl-2-associated X protein (Bax)) [108]. Similarly, Kandil et al. [109] reported that the inclusion of melatonin-loaded CH-NPs in maturation media for buffalo oocytes enhanced mitochondrial function and increased maturation rates and developmental competence compared with conventional melatonin inclusion. Notably, oocytes matured in the presence of CH-based materials consistently demonstrate higher fertilization rates, improved sperm–oocyte interactions, and enhanced zygote quality [106,107,108,109,110].

#### 5.2.3. CH and CH-Based Material Inclusion in the Culture Stage

During the IVC stage, OS and lipid peroxidation also compromise embryo viability and developmental progression. García et al. [110] reported that the addition of raw CH to porcine oocyte maturation and embryo culture media significantly increased cleavage rate, morula formation, and subsequent early embryonic development. CH-based nanocarriers have also supported embryo development during IVC. A recent study demonstrated that the supplementation of buffalo granulosa cell culture media with curcumin-loaded CH-NPs (2 µg/mL) significantly improved cell viability and increased total antioxidant capacity (TAC) and SOD1 gene expression, while reducing MDA levels and lipid peroxidation [111]. Given the critical role of granulosa cell function in determining oocyte competence, these findings support an indirect role of CH-based systems in improving embryo developmental potential during the IVC stage.

**Table 2:** Applications of chitosan (CH) and chitosan-based materials during in vitro maturation (IVM) and in vitro fertilization (IVF) of farm animals.

CH Form	Species	Cell Type	Media	Optimal Concentration/Outcomes	Proposed Mechanism of CH
CH-NPs [106,107]	Cattle (dairy cows)	Immature oocytes (COCs)	IVM (±Linoleic acid)	10 µg/mL CH-NPs in IVM + linoleic acid: ↑ Fully expanded cumulus COCs ↑ MII nuclear maturation Recovered cleavage/blastocyst rates to control Prevents the detrimental effect of linoleic acid	CH-NPs act as a chelating & antioxidant polymer Scavenge ROS Chelate lipid/metal ions to reduce OS ↓ DNA damage & restore nuclear maturation ↑ Subsequent embryo development
	Swine (sows)	Immature oocytes (COCs)	IVM	25 µg/mL CH-NPs in maturation media: ↑ Oocyte quality & developmental competence ↑ Maturation, cleavage & blastocyst rates ↑ Intracellular GSH ↑ Total cell mass (ICM: TE ratio)	CH-NPs act as an antioxidant polymer Scavenge ROS Inhibit lipid peroxidation, maintaining membrane integrity & function Protect mitochondrial function, facilitating GSH synthesis
Curcumin–CH-NPs [111]	Buffalo (cows)	IVC granulosa cells	IVC	2 µg/mL curcumin–CH-NPs in culture media: ↑ Cell viability & performance ↑ SOD1 transcription & antioxidant capacity ↓ MDA, lipid peroxidation & apoptosis	CH-NPs enhance curcumin solubility, bioavailability, stability & cellular uptake Scavenge ROS ↑ Antioxidant defense (↑ TAC, GPx & SOD1) ↓ Lipid peroxidation & MDA ↓ Low Bax expression, limiting apoptosis
Melatonin–CH-NPs [108,109]	Buffalo (cows)	Immature oocytes (COCs)	IVM	10^−9^ M melatonin–CH-NPs in maturation media: ↑ Nuclear maturation rate (MII-stage) & cumulus expansion ↑ Expression patterns associated with developmental competence & oocyte quality	CH-NPs enhance melatonin solubility, bioavailability, stability & cellular uptake Scavenge ROS ↑ SOD1 & antioxidant defense Promote oocyte developmental competence (↑ GDF9 & BMP15 expressions) ↑ Cell survival (↑ Bcl-2 & ↓ Bax)
		Immature oocytes (COCs)	IVM	10^−6^ M melatonin–CH-NPs in maturation media: ↑ Nuclear maturation (MII-stage), cleavage & blastocyst rates ↑ Mitochondrial function & membrane potential	
Raw CH [110]	Swine (sows)	Immature oocytes (COCs) & IVP embryo	IVM & IVC	50 ppm CH in maturation media: ↑ Oocyte maturation 150 ppm CH in culture media: ↑ Embryo development & morula ratio	CH exhibits antioxidant & membrane-protective effects Scavenge ROS ↓ Lipid peroxidation ↑ Oocyte & embryo quality

↑: Increase, ↓: Decrease, Bax: Bcl-2-associated X protein (pro-apoptotic), Bcl-2: B-cell lymphoma 2 (anti-apoptotic), BMP15: Bone morphogenetic protein 15, CH: Chitosan, CH-NPs: Chitosan nanoparticles, COCs: Cumulus–oocyte complexes, DNA: Deoxyribonucleic acid, GDF9: Growth differentiation factor 9, GPx: Glutathione peroxidase, GSH: Reduced glutathione, ICM: Inner cell mass, IVC: In vitro culture, IVM: In vitro maturation, MII: Metaphase II stage oocyte, MDA: Malondialdehyde, OS: Oxidative stress, ROS: Reactive oxygen species, SOD: Superoxide dismutase, TAC: Total antioxidant capacity, TE: Trophectoderm.

**Figure 3:**
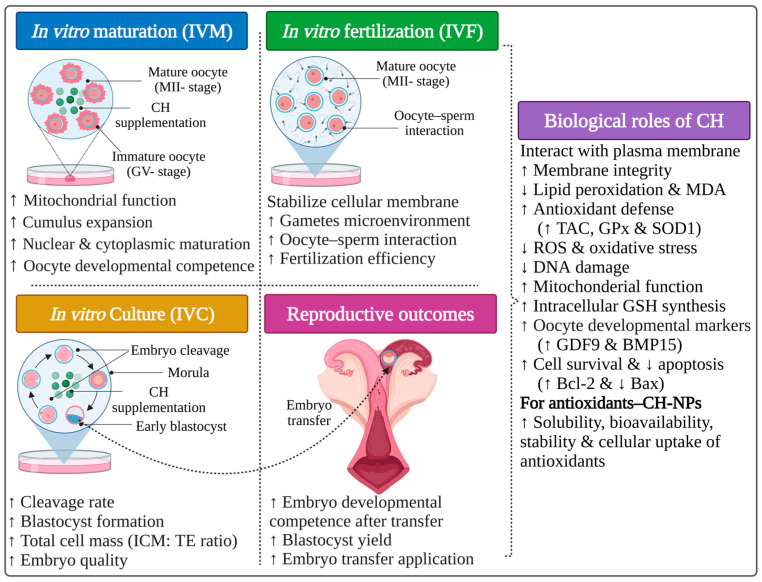
Illustration of the biological roles of chitosan (CH) and chitosan nanoparticles (CH-NPs) in farm animals and their impacts on in vitro embryo production (IVEP) stages, including in vitro maturation (IVM), in vitro fertilization (IVF), and in vitro culture (IVC), as well as subsequent reproductive outcomes following embryo transfer. ↑: Increase, ↓: Decrease.

Overall, current evidence indicates that CH and CH-based materials have beneficial effects during the IVM and IVC stages (Figure 3) by scavenging media-induced ROS, reducing OS, preserving mitochondrial function and integrity, and upregulating genes involved in cell survival and antioxidant defense. However, it is important to note that most of the studies mentioned were conducted in vitro, and few reports have evaluated pregnancy outcomes following embryo transfer. Additionally, variability in CH formulation, concentration, species, and experimental design contributes to heterogeneity in reported results. Consequently, while observations are promising at the cellular and embryonic levels under in vitro conditions, further in vivo investigations are required to confirm the beneficial impact of these materials on reproductive performance.

### 5.3. Reproductive Cycle Management (Induction of Ovulation and Estrus Synchronization)

**Figure 4:**
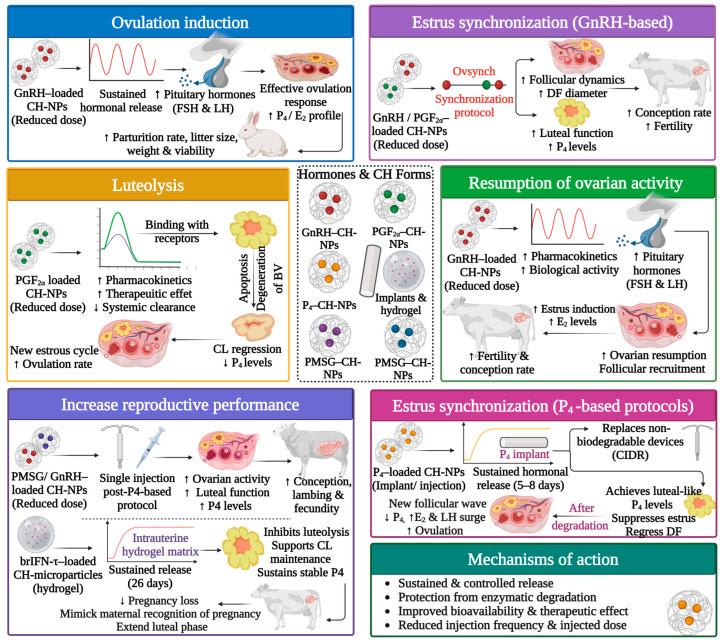
Illustration of the mechanisms of action and various biological activities of chitosan (CH)-based drug delivery systems in farm animal reproduction. The figure highlights applications, including ovulation induction, estrus synchronization using GnRH-based and progesterone (P_4_)-based protocols, luteolysis, resumption of ovarian activity postpartum, and enhancement of reproductive performance. ↑: Increase, ↓: Decrease.

CH and CH-based delivery systems have been developed as promising carriers for reproductive hormones and bioactive molecules due to their favorable physicochemical and biological properties, including biocompatibility, mucoadhesive behavior, biodegradability, and the capability to protect vulnerable compounds from enzymatic degradation [112,113]. In farm animal reproductive management, CH-based delivery systems (e.g., NPs, microparticles, hydrogels, and implants) have been widely integrated to enhance hormone bioavailability, prolong biological activity, protect against enzymatic degradation, reduce administered doses, and ultimately enhance reproductive outcomes across different farm animal species (Figure 4 and Table 3) [28].

#### 5.3.1. Hormone Delivery for Ovulation Induction

Over the last decade, several studies have investigated CH-based delivery systems as effective carriers for gonadotropin-releasing hormone (GnRH) analogues, particularly for ovulation induction in induced-ovulatory species such as rabbits. Encapsulation of GnRH analogues within CH–dextran sulfate NPs and CH–alginate NPs has enabled intravaginal administration through semen extenders, effectively replacing conventional parenteral injections [74,75,76]. These nano-formulations effectively protect GnRH from proteolytic degradation in seminal plasma and the vaginal environment, enhance mucosal adhesion, allow an initial burst of around 55% of entrapment GnRH from CH–dextran sulfate NPs during the first 5 h and then sustained hormone release, and improve GnRH bioavailability and transepithelial absorption [75]. As a result, ovulation induction and fertility have been achieved using reduced GnRH doses (from 15–25 µg down to 4 µg/doe) without affecting reproductive performance adversely [74,75,76].

Similarly, intramuscular administration of GnRH-loaded CH-TPP-NPs in rabbits, sheep, and goats markedly allowed approximately 50% reductions in the conventional hormone doses while improving ovulation induction rates, luteal function, and fertility outcomes [77,78,114]. These improvements are attributed to the enhanced pharmacokinetics and pharmacodynamics of GnRH, including initial rapid release of loaded hormones from CH-TPP-NPs of about 50% within the first 10 h, followed by sustained release over the next 20 to 30 h [77]. This improved pharmacokinetics enhanced tissue penetration and more efficient pituitary secretion of luteinizing hormone (LH) and follicle-stimulating hormone (FSH), leading to effective synchronization of ovulation and improved luteal function.

#### 5.3.2. Hormone Delivery for Estrus Synchronization and Luteolysis Control

CH-based delivery systems have also been applied for estrus synchronization protocols via encapsulation of GnRH, prostaglandin (PGF_2α_), and progesterone (P_4_). The inclusion of GnRH mixed with raw CH [82] or loaded on CH-NPs [83] as part of the Ovsynch protocol (OVS) in anestrus buffalo during low breeding season successfully induced postpartum resumption of ovarian activity, improved hormonal profiles, decreased the number of required services, and increased estrus induction and conception rates [82,83], while allowing up to 50% reduction in hormone dosage [83].

In goats, administration of GnRH analogues and PGF_2α_-loaded CH-TPP-NPs within the OVS protocol effectively enhanced ovarian activity, blood flow, steroid synthesis (estradiol (E_2_) and P_4_), and corpus luteum (CL) function during estrus synchronization [80]. Additionally, the implementation of nano-OVS incorporating nano-GnRH and nano-PGF_2α_ conjugated with CH-TPP-NPs in heat-stressed dairy cows enabled a 50–75% reduction in conventional hormone doses without compromising fertility outcomes [79]. These benefits were associated with enhanced ovarian response, increased preovulatory follicle diameter, improved luteal function, elevated P_4_ concentrations during early pregnancy, and improved overall fertility, highlighting nano-formulated hormones as promising and practical tools for enhancing reproductive performance under heat-stress conditions [79].

Moreover, CH-based systems have been used for luteolysis control via PGF_2α_ delivery. Incorporation of PGF_2α_-loaded CH-NPs into single- or double-PGF_2α_ protocols preserved luteolytic efficacy while improving hormone stability and enabling dose reduction without impairing CL regression, estrus onset, ovulation timing, or pregnancy rates in cattle [81,115]. Likewise, in infertile or repeat breeder cows, mixing PGF_2α_ with raw CH and administering it on days 5 or 11 of the estrus cycle allowed new follicular wave emergence, enhanced estrus response, and improved pregnancy and calving rates, successfully addressing infertility associated with repeated estrus or ovarian cysts [116]. These observations suggest that CH may facilitate hormone–receptor interactions and improve tissue response.

For P_4_ delivery in sheep and cows, as well as in simulated cow vaginal fluid and in vitro vaginal environments, CH microparticles and CH-based implant formulations, such as CH–polyethylene glycol (PEG) and CH–PEG–polycaprolactone (PCL) matrices, provide controlled and biodegradable alternatives to conventional intravaginal devices such as controlled internal drug release (CIDR) and P_4_-releasing intravaginal devices (PRIDs) [47,117,118]. These formulations enable sustained P_4_ release over 4 to 8 days, achieve luteal-like plasma P_4_ concentrations, and subsequently degrade without the need for device removal. Consequently, they reduce mucosal irritation, handling stress, device loss, and environmental impact [47,117,118]. Release kinetics can be optimized by adjusting particle size, crosslinking degree, and polymer composition, allowing optimization for short- or medium-term synchronization protocols. Although both CH-TPP-NPs and CH-PEG implants showed a biphasic P_4_ release pattern, their pharmacodynamic behavior differs in the mechanisms of release control. In CH-TPP-NPs, a burst phase began with ≈20 to 50% of P_4_ being released within 0 to 0.2 days (≈5 h), followed by diffusion-controlled release over 0.2 to 8 days. Increased TPP crosslinking in the formulation reduced matrix swelling and slowed diffusion, thereby slowing hormonal release during the second phase [47]. In contrast, the CH-PEG-based implants exhibited a rapid surge of P_4_ within the first day of application in the sheep vagina and stabilization at 2–3 days, followed by a marked decline by day 4, indicating matrix erosion rather than purely diffusion-controlled release [117,118].

#### 5.3.3. Drug Delivery for Enhanced Reproductive Performance

Beyond GnRH, CH-NPs have been used to deliver other reproductive bioactive components, including human chorionic gonadotropin (hCG), pregnant mare serum gonadotropin (PMSG), and NGF. Nasal administration of hCG-loaded CH-NPs in dairy cattle achieved ovulation timing, follicular development, CL formation, and estrus onset compared with intramuscular injection, emphasizing the potential of non-invasive delivery routes enabled by CH’s cationic nature and its mucoadhesive and permeability-enhancing properties [119]. Similarly, PMSG encapsulated in CH-TPP-NPs improved follicular growth, luteal function, fecundity, pregnancy, and lambing rates in synchronized ewes, while allowing an approximately 50% dose reduction [120].

CH microencapsulation has also been investigated for intravaginal delivery of NGF as a physiological alternative to GnRH in rabbit does. This formulation successfully induced ovulation by protecting NGF from enzymatic degradation, enhancing mucosal adhesion, and providing sustained release [121]. While ovulation induction was effective in receptive nulliparous does, responses were less consistent in multiparous does, highlighting the influence of animal receptivity and physiological status [121].

Recently, CH-based systems have been extended to develop reproductive biotherapeutics, for instance, interferon tau (IFN-τ), a pivotal signal for maternal recognition of pregnancy in ruminants [122]. Microencapsulation of recombinant IFN-τ (rIFN-τ) in CH particles mixed with starch–CH hydrogels enabled sustained intrauterine release for up to 26 days, maintained biological activity, inhibited luteolysis, preserved P_4_ secretion, and reduced early embryonic loss in cattle [122]. This application demonstrates the potential of CH-based systems to deliver species-specific compounds that are otherwise limited by rapid degradation.

Collectively, CH-based drug delivery systems represent promising, cost-effective, and environmentally sustainable strategies to improve reproductive efficiency while minimizing hormone use in livestock production systems. However, it is important to mention that the study designs, animal species, treatment protocols, environmental and physiological conditions, and formulation characteristics of CH differ among reports. Additionally, several studies were conducted under controlled or specific conditions or at specific physiological stages, which may limit direct applicability across different species or management systems. Therefore, large-scale experiments are needed to confirm the long-term reproductive and economic benefits of CH-based delivery systems.

#### 5.3.4. Hormonal Delivery for Male Reproductive Management

Although less investigated in males than in females, CH-based delivery systems show substantial promise for male reproductive management. A recent study in vertebrate models (*Labeo rohita*) demonstrated that the full dose of CH-conjugated GnRH analogues significantly improved testicular activity and histoarchitecture, boosted sperm quality, and strengthened endocrine responses. These effects were accompanied by upregulation of gene expression related to the β-subunits of pituitary gonadotropins (FSHβ and LHβ) and their respective receptors (FSHr and LHr) in the testes [84]. These observations suggest potential applications of CH-based delivery systems in farm animals for improving semen quality and overall reproductive efficiency in males.

**Table 3:** Applications of chitosan (CH) and chitosan-based materials as a drug delivery system in farm animals.

CH Form	Encapsulated Drug	Species	Aim of the Application	Route	Optimal Dose/Outcomes	Proposed Mechanism of CH
CH–dextran sulfate NPs (or) CH–alginate NPs [74,75,76]	GnRH (buserelin acetate)	Rabbit (does)	Ovulation induction	Intravaginal (in semen extender)	4 µg/doe GnRH-CH-NPs (dextran sulphate) ↑ Fertility & prolificacy ↓ GnRH dose by 20% without affecting fertility	Encapsulated GnRH in CH-NPs: Protects GnRH from enzymatic degradation in semen & vagina ↑ GnRH bioavailability at a lower dose Provides a mucoadhesive carrier in the vaginal mucosa to prolong local residence time Controls hormonal release ↑ GnRH absorption across vaginal epithelium Allows ovulation induction Achieves normal fertility with reduced dose
			Ovulation induction	Intravaginal (in semen extender)	4 µg/doe GnRH-CH-NPs (alginate or dextran sulphate): ↑ Fertility & prolificacy ↓ GnRH dose from 15–25 µg to 4 µg without affecting fertility Avoiding parenteral injections	
CH-TPP-NPs [77,114]	GnRH (buserelin acetate)	Rabbit (does)	Ovulation induction	Intramuscular or intravaginal infusion	0.4 µg/doe GnRH-CH-NPs (i.m.) Stimulates earlier LH surge ↑ Ovulation points, conception, ovulation rate & E_2_/P_4_ profiles ↑ Parturition rate, litter size, weight & viability ↓ GnRH dose by 50%	Encapsulated GnRH in CH/CH-NPs: GnRH protected from enzymatic degradation in blood Prolongs effective half-life Facilitates passage across biological barriers & improves GnRH bioavailability Enables lower doses to reach the pituitary effectively Enhances GnRH delivery, explains earlier LH surge & adequate ovulatory response (in rabbit) ↑ LH/FSH release, more ovulations (↑ CLs number), luteal function (↑ P_4_) Supports embryo survival (in goat) Enhances the therapeutic effect of GnRH in anestrus buffalo under heat stress ↑ Pharmacokinetics & pharmacodynamics of GnRH explain why reduced dose yields better fertility (in rabbit, buffalo & goat)
	GnRH (gonadorelin)	Goat (does)	Increase reproductive performance	Intramuscular injection	12.5 µg GnRH-CH-NPs ↓ Ovulation rate, prolificacy & P_4_/E_2_ patterns ↓ Pregnancy loss & GnRH dose by 75% without affecting fertility	
CH solution [82]	GnRH (buserelin acetate)	Buffalo (cows) (Anestrus)	Ovarian resumption	Intramuscular injection	0.02 mg/dose GnRH-conjugated CH within OVS protocol on D0 & D9: ↑ Ovarian resumption, estrus induction & conception rates ↑ Diameter of DF ↑ P_4_ & E_2_ patterns	
CH-TPP-NPs [78,79,80,81,83,115]	GnRH (gonadorelin)	Buffalo (cows) (Anestrus)	Ovarian resumption	Intramuscular injection	125 µg GnRH-CH-NPs within OVS protocol on D0 & D9: ↑ Ovarian resumption, estrus induction & conception rates ↑ Follicles number & DF diameter ↑ P_4_ & E_2_ patterns & pregnancy ↓ S/C & GnRH dose by 50%	
		Sheep (ewes) (Anestrus)	Estrus & ovulation induction	Intramuscular injection	50 µg GnRH-CH-NPs instead of eCG in P_4_-based protocol: Similar ovarian activity ↑ P_4_ levels during the early luteal phase & early pregnancy ↑ Conception, lambing & fecundity	Encapsulated GnRH in CH-NPs: Enhances GnRH delivery Supports better luteinization and CL function ↑ P_4_ levels and better fertility
	GnRH (buserelin acetate) & PGF_2α_	Cattle (dairy cows)	Estrus synchronization	Intramuscular injection	2.5 µg GnRH-CH-NPs & 125 µg PGF_2α_-CH-NPs in OVS protocol: ↑ Follicular dynamics & DF diameter ↑ P_4_ level & pregnancy rate ↓ GnRH dose by 50–75%	Encapsulated GnRH & PGF_2α_ in CH-NPs: Enhances GnRH & PGF_2α_ delivery ↑ Ovsynch protocol efficiency ↑ Ovarian blood flow & follicular growth ↑ Luteal function ↑ Pharmacokinetics & pharmacodynamics explain why 25%/50% of GnRH & PGF2α doses yield better fertility than full conventional doses
	GnRH (ovurelin) & PGF_2α_	Goat (does)	Estrus synchronization	Intramuscular injection	25 µg GnRH-CH-NPs & 62.5 µg PGF_2α_-CH-NPs in OVS protocol: ↑ Ovarian blood flow & dynamics ↑ CL diameter & function (P_4_ level) ↓ GnRH dose by 50%	
	PGF_2α_	Cattle (dairy cows)	Estrus synchronization & luteolysis	Intramuscular injection	Using PGF_2α_-CH-NPs in double-PGF_2α_ protocol Similar ability to CL regression Maintains comparable efficacy to conventional PGF_2α_	Encapsulated PGF_2α_ in CH-NPs: Provides a nano-sized, positively charged carrier ↑ Stability & interaction with tissue while preserving biological activity Maintains PGF_2α_ pharmacodynamics Similar CL regression, P_4_ reduction Similar estrus rate & ovulation parameters compared with conventional PGF_2α_
		Cattle (beef cows)	Estrus synchronization & luteolysis	Intramuscular injection	Using PGF_2α_-CH-NPs in single/double-PGF_2α_ protocol: ↑ Luteolysis & estrus response ↑ Pregnancy rate	
Raw CH [116]	PGF_2α_	Cattle (dairy cows) (Infertile)	Estrus & ovulation induction	Intramuscular injection	PGF_2α_ mixed with CH on day 5 or day 11 of the estrous cycle: Successfully treated infertility (repeat breeder/cystic ovary) ↑ Estrus, pregnancy & calving rates	PGF_2α_ mixed with CH polymer: ↑ Effectiveness of PGF_2α_ in repeat breeder & cystic ovarian syndrome Binds to CL receptors & induces luteolysis ↓ P_4_, allowing new follicular wave & estrus
CH-TPP-NPs [47,119,120]	hCG (choluron)	Cattle (dairy cows)	Ovulation induction	Nasal spray	1000 IU of hCG-CH-NPs is effective as an i.m. injection. No adverse impact on ovulation time, follicle size, CL regression & estrus onset	hCG encapsulated in CH-NPs: Protects hCG from enzymatic degradation Provides a mucoadhesive carrier in the nasal mucosa to prolong local contact time Facilitates hormone absorption by opening tight junctions between epithelial cells
	PMSG	Sheep (ewes)	Increase reproductive performance	Intramuscular injection	300 IU PMSG-CH-NPs after synchronization by short-term P_4_ protocol ↑ Fecundity, pregnancy & lambing ↑ Large follicles number & diameter ↑ CLs number & P_4_ concentration ↓ PMSG dose by 50%	PMSG encapsulated in CH-NPs: Protects PMSG from enzymatic degradation & improves stability ↑ Bioavailability and cellular uptake 50% reduction in conventional dose without affecting fertility
	P_4_	Cattle (dairy cows)	Estrus synchronization	Parenteral injection	A single injection of P_4_-CH-NPs: Provides sustained P_4_ within 5–8 days ↓ Mucosal irritation, handling & device loss	P_4_ encapsulated in CH-NPs: Creates a stable biodegradable matrix Provides sustained hormonal release by an initial burst from the particle’s surface, then diffusion-controlled release from CH-NPs Replace non-biodegradable intravaginal devices (CIDR, PRID, etc.)
CH-PEG matrix [117]	P_4_	Sheep (ewes)	Estrus synchronization	Intravaginal implant	Using P_4_-CH-PEG matrix as an intravaginal implant: Delivers P_4_ for 4 days at luteal levels & then fully degrades Offers a biodegradable alternative to conventional devices	P_4_-CH-PEG matrix acts as a hydrophobic, biodegradable P_4_ depot Water uptake after insertion causes swelling, erosion & controlled P_4_ release Achieves luteal-like P_4_ levels that suppress estrus & regress DF Stimulates the emergence of new follicles Implant degradation reduces P_4_, allowing E_2_ rise, LH surge & ovulation
CH microparticles in starch–CH hydrogel [122]	brIFN-τ	Cattle (beef cows) & sheep (ewes)	Inhibit luteolysis & support CL maintenance	Intrauterine deposition	Using brIFN-τ-CH microparticles in starch–CH hydrogel: Provides a long-acting & safe delivery of brIFN-τ Boosts induction of IFN-stimulated genes (PKR, OAS1, OAS2) Inhibits luteolysis, supports CL maintenance & sustains stable P_4_ ↓ Pregnancy loss	Microencapsulated brIFN-τ in CH: Protects brIFN-τ from rapid degradation Enables sustained release up to 26 days Hydrogel matrix prolongs uterine residence Maintains an anti-luteolytic intrauterine environment by mimicking maternal recognition of pregnancy and extending the luteal phase
CH-NPs [121]	rrβ-NGF	Rabbits (does)	Ovulation induction	Intravaginal in semen extender	0.5 µg/doe rrβ-NGF-CH microparticles 30 min before AI: ↑ Ovulation & pregnancy rate in nulliparous, but not multiparous rabbit does	NGF encapsulated in CH microparticles: NGF protected from enzymatic degradation Provides a mucoadhesive carrier in the vagina mucosa to prolong local contact time Provides sustained release over a short time NGF could induce GnRH release from hypothalamic neurons & induce ovulation

**↑:** Increase, ↓: Decrease. AI: Artificial insemination, brIFN-τ: Bovine recombinant interferon tau, CH: Chitosan, CH-NPs: Chitosan nanoparticles, CIDR: Controlled internal drug release, CL: Corpus luteum, DF: Dominant follicle, E_2_: Estradiol, eCG: Equine chorionic gonadotropin, FSH: Follicle-stimulating hormone, hCG: Human chorionic gonadotropin, i.m.: Intramuscularly, GnRH: Gonadotropin-releasing hormone, LH: Luteinizing hormone, OAS: Oligoadenylate synthetase, OVS: Ovsynch, P_4_: Progesterone, PEG: Polyethylene glycol, PGF_2α_: Prostaglandin, PKR: Protein kinase R, PMSG: Pregnant mare gonadotropin, PRID: Progesterone-releasing intravaginal device, rrβ-NGF: Rabbit recombinant β-nerve growth factor, TPP: Sodium tripolyphosphate.

### 5.4. Uterine Health (Disease Management, Preventive Application and CH-Based Delivery Systems)

#### 5.4.1. Antimicrobial Activity and Uterine Microbiome Modulation

CH microparticles exhibit broad-spectrum antimicrobial activity against major uterine pathogens, including *Escherichia coli* (*E. coli*), *Fusobacterium necrohporum*, *Arcanobacterium (A) pyogenes*, *Bacteroides* spp., and other Gram-positive and Gram-negative bacteria [46,56,123]. This broad antimicrobial activity of CH may be attributed to the electrostatic interactions between cationic CH and anionic outer membrane components such as LPSs and outer membrane protein A (OmpA), leading to bacterial cell death without inducing antimicrobial resistance or prophage activation [46,123].

An in vitro study investigated the antibacterial efficacy of CH-NPs against pathogens isolated from dairy cows with subclinical endometritis [124]. The results revealed that treatment with CH-NPs (0.5–2.0%) markedly reduced *Pseudomonas aeruginosa*, *Klebsiella* spp., *Staphylococcus aureus*, and *Bacillus* spp., while having a limited or no effect on *E. coli* and *A. pyogenes.* These observations indicate that the effectiveness of CH-NPs depends on both pathogen sensitivity and treatment concentration [124].

Further in vivo investigations have demonstrated that intrauterine administration of CH microparticles can reduce uterine pathogen load and alter the uterine microbial population by decreasing pathogenic taxa, such as *Fusobacteriaceae* and *Bacteroidaceae*, thereby shifting the uterine microbiota toward a healthier profile [123]. However, subsequent field and microbiome-focused studies revealed inconsistent and, in some cases, detrimental impacts. In cows with subclinical endometritis, intrauterine application of CH microparticles slowed the transition of the uterine microbiome toward a healthier state, increased total bacterial loads, and maintained the persistent dominance of *Fusobacterium* spp. compared with ceftiofur-treated cows [125]. These findings suggest that CH efficacy may be influenced by dosage, pathogen sensitivity, and microbiome dynamics.

#### 5.4.2. Clinical Efficacy in Metritis and Endometritis

The therapeutic efficacy of CH microparticles for treating metritis remains controversial. Although early experiments reported reductions in uterine infection following postpartum intrauterine administration of CH microparticles [47,116], large randomized controlled trials showed that CH microparticles did not improve cure rates for metritis and were inferior to ceftiofur with respect to clinical recovery, milk yield, survival, and fertility [126].

In some cases, CH microparticle treatment was associated with increased culling risk, delayed conception, and behavioral alterations, such as decreased rumination, suggesting an exacerbation of uterine or systemic inflammation [126,127]. Microbiome and behavioral data suggest that these adverse outcomes may result from excessive or improperly directed local immune stimulation rather than insufficient antimicrobial activity alone [125,127]. At higher concentrations of 0.6%, CH microparticles may intensify uterine inflammation, delay tissue recovery, and disrupt microbial stabilization, ultimately impairing reproductive performance [125,126].

In contrast, CH solutions administered by intrauterine infusion at lower concentrations showed more favorable outcomes in cases of clinical and subclinical endometritis. In postpartum cows with endometritis, CH solution significantly reduced polymorphonuclear leukocyte (PMN) proportions and accelerated uterine recovery without adverse effects on fertility [128]. Collectively, these findings suggest that the formulation, dose, and physicochemical properties of CH critically influence uterine responses and therapeutic success.

#### 5.4.3. Preventive Applications and Reproductive Outcomes

Preventive application of CH in cows at high risk for metritis yielded mixed results. Intrauterine infusion of CH microparticles shortly after calving modestly reduced the incidence of metritis during the first 7 days in milk. However, this protective effect was not sustained beyond the early postpartum period [55]. Notably, CH microparticles did not markedly alter systemic inflammatory or metabolic markers, indicating limited physiological impact at the tested dose and duration [55].

Beyond infection control, intrauterine infusion of CH solution has been shown to improve reproductive performance. In anestrus and postpartum cows, CH infusion before estrus or ovulation synchronization substantially increased the conception rate compared with hormonal protocols applied without previous treatment [129]. Similarly, intrauterine CH infusion in beef cows shortened the interval to the first postpartum estrus and improved reproductive efficiency, likely by accelerating uterine involution and restoring a healthy uterine environment [130].

#### 5.4.4. Chitosan-Based Delivery Systems

Recent reports have focused on CH-based hydrogels and nano-delivery systems designed to overcome the limitations of conventional intrauterine treatments. For instance, CH–polyvinyl alcohol hydrogels loaded with the antibiotic ofloxacin have achieved sustained drug release, enhanced uterine retention, potent antibacterial activity, and marked clinical recovery from metritis in cattle, while minimizing systemic exposure and adverse effects [35]. Also, in an experimental rodent model of endometritis, thermosensitive injectable CH-based hydrogels loaded with bioactive compounds, such as berberine and carvacrol, effectively reduced bacterial load and uterine inflammation [34]. Moreover, CH gels containing polymeric nano-capsules have shown strong mucoadhesive properties and enhanced penetration of hydrophobic compounds across vaginal and uterine mucosa when applied ex vivo, supporting their potential as localized drug delivery vehicles for uterine therapy [68].

**Figure 5:**
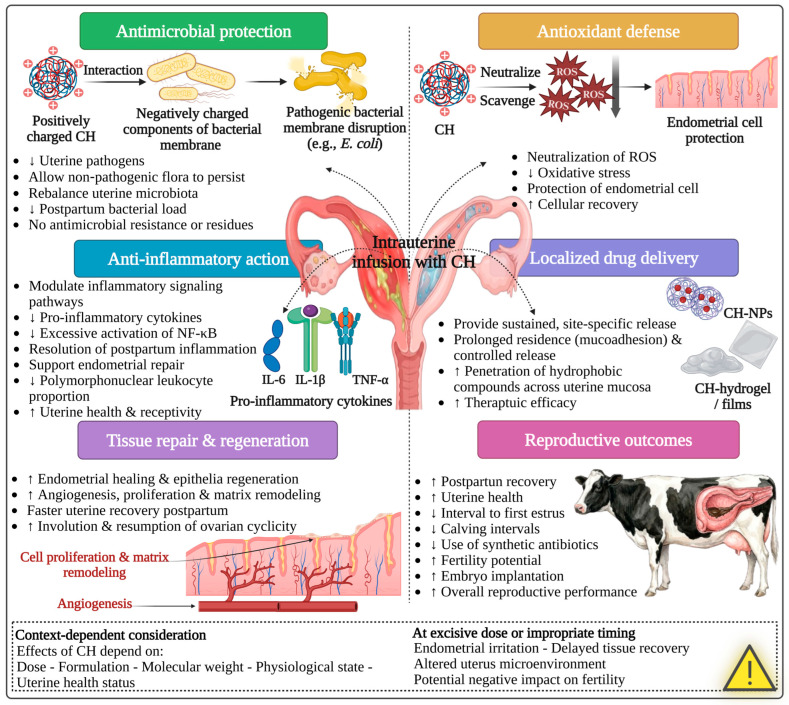
Representation of the roles of intrauterine infusion with chitosan (CH) and chitosan-based materials in the uterine health of farm animals. The figure illustrates the essential effects of the materials, including antimicrobial protection, antioxidant defense, anti-inflammatory action, localized drug delivery, and postpartum tissue repair and regeneration, as well as their overall impact on reproductive outcomes. The figure also highlights the context-dependent nature of their effects and the potential adverse impacts associated with excessive dosages. ↑: Increase, ↓: Decrease.

Overall, the available evidence highlights the substantial antimicrobial and immunomodulatory potential of CH for the management of uterine health (Table 4 and Figure 5). However, the results across available studies are obviously heterogeneous, with clinical efficacy highly influenced by formulation, dose, timing, and disease status. While some studies have reported improvements in uterine recovery and subsequent reproductive performance, other studies have reported limited or adverse clinical outcomes under certain conditions. Therefore, further large-scale experiments are necessary to define optimal protocols, doses, and physicochemical properties to clarify its therapeutic effect.

### 5.5. Direct Effect of Dietary Chitosan on Reproductive Performance of Farm Animals

Several studies demonstrate that dietary CH and CH-based materials exert direct and substantial effects on reproductive performance in farm animals (Table 5). These effects include gametogenesis, reproductive behavior, endocrine regulation, pregnancy outcomes, placental function, embryonic and fetal survival, and offspring viability. These effects are reported across different species and reproductive stages, supporting the role of CH as a functional feed additive with reproductive-modulating properties.

#### 5.5.1. Male Reproductive Function

In male animals, dietary CH and CH-based materials have been shown to directly enhance semen quality and fertility outcomes. In heat-stressed rabbit bucks, the inclusion of astaxanthin-loaded CH-NPs (100–150 mg/kg) significantly enhanced sperm motility, viability, concentration, and acrosomal integrity, alongside significant improvements in testicular function, histoarchitecture, and physiological responses [131]. Additionally, molecular analyses further demonstrated that astaxanthin-loaded CH-NPs bind to crucial antioxidant-related proteins, including GPx, SOD, and nuclear factor erythroid 2-related factor (Nrf2), supporting enhanced antioxidant signaling critical for maintaining sperm function under heat stress. These improvements translated into markedly higher pregnancy rates in females mated with treated males [131].

Similarly, dietary supplementation of raw CH (2.5 mg/kg concentrate) in goat bucks fed a high-fat diet significantly improved ejaculate volume, sperm motility, viability, concentration, and DNA integrity while reducing sperm abnormalities, necrosis, and lipid peroxidation in seminal plasma [132]. These reproductive enhancements were associated with increased antioxidant enzyme activities (GSH, CAT, and GPx) and increased testosterone concentrations, emphasizing direct modulation of testicular redox balance and steroidogenic activity, along with mitigation of the adverse impacts of high-fat diets on hematological parameters [132,145]. Comparable improvements were obtained in rabbit bucks fed raw CH, which enhanced sexual behavior, libido, semen quality, and overall reproductive efficiency, with optimal responses observed at dietary levels of 0.2–0.4 mg/kg [133].

These reports confirm that CH can directly support spermatogenesis, sperm viability and integrity, endocrine profile, and matting efficiency, particularly under oxidative, thermal, or nutritional stress.

#### 5.5.2. Female Reproductive Function and Pregnancy Outcomes

In females, dietary CH supplementation has demonstrated direct effects on ovarian activity, estrus behavior, conception, gestation, and parturition. In rabbit does, inclusion of raw CH at 0.2 mg/kg diet markedly increased ovarian activity, the number of matured follicles, receptivity rate, conception rate, and parity number while reducing receptivity duration and kindling interval [134].

In swine, COSs have been shown to have robust direct effects on reproductive performance during gestation. Dietary supplementation with 100 mg/kg COSs increased fetal survival rate, reduced stillbirths and mummification, and increased litter size and birth weights in multiparous sows [135,137,138]. These reproductive benefits were accompanied by enhanced maternal antioxidant capacity, increased immunoglobulin concentrations, and improved placental angiogenesis and nutrient transport. Molecular analyses revealed upregulation of placenta and fetal genes involved in growth and immune response, including leptin, vascular endothelial growth factor A (VEGF-A), signal transducer and activator of transcription 3 (STAT3), transforming growth factor beta (TGF-β), and fibroblast growth factor receptor 2 (FGFR2) [137]. Further transcriptomic profiling of sow ovaries revealed that COS supplementation directly modulated genes associated with P_4_-mediated oocyte maturation, oocyte meiosis, cell cycle regulation, and metabolic pathways, providing molecular markers that COSs improve fertility at the ovarian follicular level [140]. Moreover, metabolic and biochemical analyses revealed that COSs alter the antioxidant, immune, and metabolic profiles of amniotic fluid, creating an optimal intrauterine environment for embryonic survival and development [141].

Beyond ovarian level effects, COSs also directly improved placental function and fetal programming. Addition of COSs during late gestation boosted placental antioxidant defense by activating endogenous antioxidants, such as SOD, thereby mitigating oxidative damage and protecting maternal and placental tissues [42]. Also, COSs reduced inflammatory signaling, activated mechanistic target of rapamycin (mTOR) pathways, and increased the expression of amino acid and glucose transporters (GLUT1, GLUT3, and SNATs), all of which support fetal growth and reduce the incidence of intrauterine growth restriction (IUGR) [42,139]. These placental adaptations translated into lower fetal loss, healthier offspring, and improved postnatal performance.

Additionally, COS supplementation during late gestation and lactation phases consistently increased milk composition, colostrum immunoglobulin content, and offspring growth rates in pigs [42,136,138,142,143,144]. These maternal effects directly contributed to improved piglet survival, intestinal development, immune competence, glucose homeostasis, and antioxidant capacity during early life. Similar benefits were observed with raw CH supplementation during the transition period, which improved maternal performance, milk production, and offspring growth [146]. In goats, dietary inclusion of CH at optimal concentrations (0.4 mg/kg dry matter (DM)) increased serum glucose and urea concentrations, indicating altered maternal energy and nitrogen metabolism that supports physiological requirements during late gestation and early lactation. Correspondingly, increased milk yield and composition and enhanced offspring growth were observed for CH-supplemented dams compared with non-supplemented dams [146].

Overall, these outcomes highlight the role of CH and its derivatives in extending reproductive success beyond conception, positively influencing placental efficiency, fetal development, offspring viability, and postnatal growth and productivity.

## 6. Indirect Implications of Dietary Chitosan for Reproductive Function

**Figure 6:**
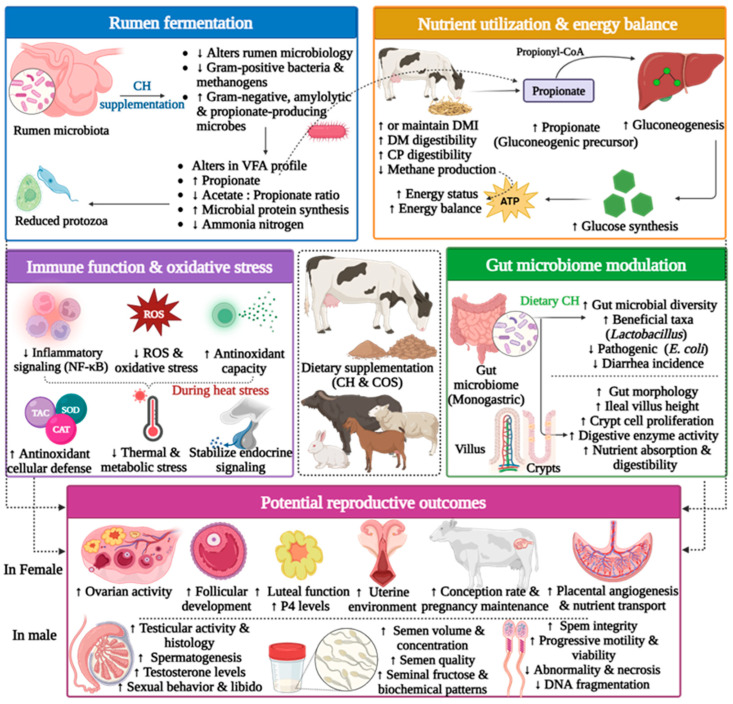
Depiction of the indirect effects of dietary chitosan (CH) and chitosan oligosaccharides (COSs) on potential reproductive outcomes in male and female farm animals. CH and COSs can influence reproductive performance indirectly through modulating rumen fermentation and microbiota, improving nutrient utilization and energy balance, regulating gut microbiome and immune function, and reducing oxidative and heat stress. ↑: Increase, ↓: Decrease.

CH and CH-based materials have been widely used as functional feed additives in farm animal nutrition due to their ability to modulate rumen fermentation, nutrient utilization, metabolic efficiency, immune status, and OS elimination [4,24]. Although many studies have not directly measured reproductive traits, the consistent improvements observed in energy metabolism, inflammatory status, gut health, and physiological homeostasis strongly suggest indirect benefits for reproductive performance, as shown in Figure 6. These are particularly relevant in high-producing animals, where fertility is tightly linked to metabolic balance and environmental conditions [147,148].

### 6.1. Gut Microbiota Modulation and Fermentation Patterns

In monogastric species, CH and CH-NPs have been applied to modulate gut microbiota and enhance intestinal health. In weaned piglets, CH-NPs (400 mg/kg) improved gut morphology and microbial diversity, increased beneficial taxa such as *Prevotellaceae* and *Ruminococcaceae*, and decreased pathogenic *Firmicutes* (*Clostridiaceae*), subsequently reducing diarrhea incidence [149]. Similarly, supplementation with low-MW COSs (150 mg/kg diet) enhanced ileal villus height, crypt cell proliferation, nutrient absorption, and digestibility [43].

In rabbits, CH-NPs used as a nanocarrier for carvacrol (400–500 mg/kg) improved cecal microbiota composition by increasing *Lactobacillus* abundance and reducing pathogenic bacteria, while increasing digestive enzyme activity and nutrient utilization [150]. Additionally, dietary inclusion of raw CH (1000 mg/kg diet) in male rabbits improved growth performance and reduced the counts of pathogenic cecal bacteria, including coliforms, *E. coli*, and *Salmonella* spp., and it also enhanced intestinal ultrastructure, including villus height and width and crypt depth [37].

In ruminants, CH supplementation has been shown to change rumen microbiology and fermentation patterns. A RUSITEC in vitro study demonstrated that CH addition at 5% markedly altered rumen microbial communities by reducing overall diversity, suppressing fibrinolytic taxa, and enriching amylolytic and propionate-producing populations. These alterations enhanced fermentation efficiency, increased propionate production, improved nitrogen utilization, and reduced methane production [38]. In agreement with these findings, CH supplementation in cows and goats selectively inhibits Gram-positive bacteria, protozoa, and methanogens, while promoting Gram-negative bacteria, amylolytic microbes, and propionate-producing microbes [40,151]. These shifts increased propionate concentration and total volatile fatty acids (VFAs), enhanced microbial protein synthesis, and reduced ammonia nitrogen (NH_3_-N) and butyrate counts, without adversely impacting dry matter intake (DMI) [40,41,151,152].

In lactating cows, dietary CH supplementation at 135 mg/kg body weight (BW)/day induced slight shifts in rumen fermentation and microbiome composition toward a more propionate-oriented profile, with increased abundance of *Anaeroplasma* and trends toward reduced fibrolytic fungi, protozoa, and methanogenic archaea (*Methanosphaera*). However, at this dose, changes were insufficient to significantly reduce methane emission or improve milk yield or composition [39].

Mechanistically, the polycationic nature of CH allows interaction with bacterial cell membranes, modulation of fermentation substrates, and enhancement of mucosal adhesion and intestinal absorption [43,149,150]. These fermentation alterations increase propionate and reduce acetate production, thereby improving glucogenic efficiency, reducing methane-associated energy loss, and supporting hepatic glucose synthesis [40,41,151,152]. Propionate is a key gluconeogenic precursor closely linked to energy balance and directly influences ovarian function, estrus resumption, and conception. Consequently, improved rumen fermentation and energy status are relevant for reproductive performance, especially during the postpartum period when a negative energy balance commonly delays ovulation and reduces fertility [153,154,155].

Moreover, CH-based delivery systems could protect sensitive substrates from rapid degradation in the rumen. For instance, using alginate–CH beads encapsulating glycerol prevented immediate rumen fermentation, enabling controlled release and boosting post-ruminal absorption through ionic gelation, which stabilized the substrate and pH-responsive mechanisms [156]. Stabilized fermentation patterns and improved glucogenic supply further improve metabolic signals such as insulin and insulin-like growth factor 1 (IGF-1), which are essential for follicular development and early embryonic survival [156,157,158].

### 6.2. Nutrient Utilization

CH has been shown to improve protein and fiber digestibility and overall nutrient utilization. In mid-lactation cows, supplementation with 100–150 mg/kg BW of CH increased crude protein digestibility by ~3%, DM digestibility by ~2%, and reduced fecal nitrogen excretion [159]. Goats receiving 360 mg/kg DM of CH exhibited improved organic matter, crude protein, and neutral detergent fiber digestibility, along with increased ruminal propionate and decreased fibrolytic microbial populations [40,41]. Similar benefits were observed in buffaloes, especially when combined with CH (150 mg/kg BW) with whole raw soybeans. This combination enhanced VFA production, nutrient retention, and propionate concentration without decreasing DMI, apparent nutrient digestibility, rumen kinetics, or microbial protein synthesis [160,161]. Also, supplementation with extracted CH at 2% of DM increased propionate concentration and the acetate-to-propionate ratio, while reducing methane production in lactating dairy cows under a tropical climate [162]. Further, extracted CH showed better economic efficiency than commercial CH, while contributing to shrimp waste recycling and environmental sustainability [162].

Improved nutrient utilization supports body condition and energy status, reducing postpartum anestrus and improving conception rates by maintaining glucose and IGF-1 levels. In addition, enhanced nutrient retention promotes energy partitioning towards the reproductive process, particularly during lactation when energy demands are high [163,164].

### 6.3. Metabolic Efficiency

CH supplementation improves feed conversion efficiency and energy utilization. In early lactation ewes, the inclusion of 1.2% DM CH maintained milk yield despite reduced DMI, showing increased plasma glucose and blood urea nitrogen and improved milk/DMI and fat-corrected milk/DMI ratios [165]. In mid-lactation cows, CH increased milk protein and lactose yields without affecting DMI, indicating enhanced metabolic efficiency [159,166].

These effects are attributed to increased propionate-driven glucose production, enhanced ruminal protein escape, and ionophore-like antimicrobial activity that shifts rumen fermentation toward more energetically efficient pathways [40,41,165]. Improved energy efficiency reduces negative energy balance, moderates metabolic stress, and supports ovarian cyclicity and uterine health postpartum [158,164].

### 6.4. Immune Function

CH enhances immune responses in both monogastric and ruminant species. In piglets, CH-NPs (400 mg/kg) increased immunoglobulin (IgA and IgM) and complement C3 and C4 levels and improved resistance to LPS challenge [149]. In rabbits, CH-NPs elevated IgM, IgA, and complement activity, thereby enhancing systemic immunity [150]. Raw CH supplementation (1000 mg/kg diet) in male rabbits increased immune function by increasing hematocrit percentage, serum lysozyme activity, IgM, and complement C3 concentrations [37]. Similar immunomodulation effects were observed with carvacrol-loaded CH-NPs (400–500 mg/kg) in the male rabbit diet [150].

Boosted immune status reduces inflammation and OS, thereby supporting better ovarian activity, uterine health, embryonic implantation, and overall reproductive efficiency [167].

### 6.5. Oxidative Balance and Heat-Stress Resistance

#### 6.5.1. CH Applications for Heat Stress and Mechanisms

CH and CH-based materials have also been used as nutritional supplementation strategies to alleviate heat stress in farm animals by targeting oxidative, inflammatory, immune, and metabolic disturbances induced by elevated ambient temperatures. In multiparous Holstein cows, dietary CH increased antioxidant enzyme activities, including SOD and GPx, while reducing MDA and NO levels. These alterations were accompanied by suppression of nuclear factor kappa-B (NF-κB) and induced NO synthase (iNOS) expression, indicating reduced oxidative and inflammatory stress [166].

Similarly, in rabbits, carvacrol-loaded CH-NPs (400–500 mg/kg) markedly reduced hepatic enzyme activities (ALT, AST, and LDH) and lipid peroxidation while increasing TAC and antioxidant enzyme activities (CAT, SOD, and GPx) [150]. Berberine-loaded CH-NPs further improved growth performance, feed conversion, lipid profile, antioxidant enzyme activity, lysozyme levels, immune markers, and thermoregulatory responses in heat-stressed male rabbits, and reduced inflammatory cytokines and OS markers [37].

Under severe heat-stress conditions (temperature–humidity index; THI = 30), dietary CH supplementation and nano-formulated CH systems consistently improved growth rate, feed efficiency, physiological stability, and organ health in male rabbits [168,169,170]. For example, raw CH (0.2 g/kg diet) in combination with 1 mL silver NPs (Ag-NPs) and 100 mg silymarin mixture enhanced antioxidant capacity, reduced lipid peroxidation and inflammatory cytokines, improved hematological and metabolic indices, and eliminated liver histopathological damage without affecting feed intake [170].

Superior efficacy was achieved when CH-NPs were used as carriers for bioactive compounds such as berberine or piperine, which significantly enhanced bioavailability and stress-mitigating potency. Berberine-loaded CH-NPs (40–50 mg/kg diet) reduced rectal temperature and respiratory rate and suppressed NF-κB signaling, OS, and pro-inflammatory cytokines while increasing antioxidant enzyme activity, immunoglobulin levels, NO production, growth performance, and liver integrity [168]. Similar improvements, along with reductions in thermal-stress biomarkers, including SOD, GPx, CAT, interleukins (IL-4 and IL-6), IFN-γ, and tumor necrosis factor alpha (TNF-α), were observed following the inclusion of piperine-loaded CH-NPs (100–150 mg/kg diet) [169].

These favorable effects are mainly mediated by inhibition of NF-κB signaling, ROS scavenging, enhancement of endogenous antioxidant defense (SOD, CAT, and GPx), activation of Nrf2-regulated pathways, improved intestinal absorption via nano-delivery systems, and stabilization of metabolic and immune functions [168,169,170]. Collectively, the results reveal CH and CH-based materials as safe, multifunctional, and sustainable nutritional tools for improving heat-stress resilience and maintaining productivity under thermal-stress conditions.

#### 6.5.2. Implications for Reproductive Function Under Heat Stress

Notably, heat stress is a significant trigger of OS, as elevated ambient temperatures increase ROS production while overwhelming endogenous antioxidant defense [171]. Consequently, enhancing systemic redox balance through CH supplementation may support reproductive cell survival, embryo development, and the integrity of reproductive tissues, particularly under conditions of heat-induced oxidative damage.

Mitigation of OS has significant implications for reproductive function under thermal challenge. Heat-stress-induced oxidative imbalance disrupts endocrine regulation and follicular growth, reduces estrus expression, impairs oocyte and embryo quality, and reduces conception rates in farm animals [172,173]. Elevated THI alters the hypothalamic–pituitary–gonadal axis, compromises oocyte maturation and developmental competence, and contributes to lower conception rates [174,175]. Additionally, high THI suppresses estrus behavior and reduces pregnancy rates by impairing steroid hormone production and follicular function, while increasing embryonic loss and reducing embryonic development [172,176]. In this context, integrating CH and CH-based materials as nutritional interventions to mitigate heat stress may help maintain the estrous cycle, preserve ovarian function, support a healthier follicular environment, and improve fertilization, embryo survival, and embryonic development outcomes during heat stress.

## 7. Safety, Regulatory, and Economic Considerations

Although the Food and Drug Administration (FDA) has identified CH and its derivatives as generally recognized as safe (GRAS) for different applications associated with food and pharmaceuticals [44] and the European Food Safety Authority (EFSA) has recognized it as a non-toxic substance for animals and humans [177], some safety considerations remain relevant for farm animal applications. The biological efficacy and safety profiles of CH and CH-based materials are influenced by MW, DD, formulation process, dosage, route of administration, animal species, and physiological status. Dose-dependent effects should be carefully taken into account, as higher concentrations may alter cellular interactions or physiological responses [178]. Additionally, although the CH is known to be biocompatible and biodegradable, its potential for immunogenic reactions or immunomodulation, especially with long-term use, warrants further investigation. Data on long-term reproductive safety, especially under commercial farm conditions, remain limited. Moreover, potential residues in animal products such as milk and meat have not been fully demonstrated.

From a practical perspective, large-scale implementation of CH-based materials requires data on optimal doses, withdrawal periods, and methods for controlling CH formulations. Furthermore, the production costs of CH-based materials and economic feasibility analyses comparing them with conventional hormones, antibiotics, or other treatments remain limited and require further evaluation to determine their commercial viability.

## 8. Conclusions

Overall, CH and CH-based materials, owing to their unique characteristics such as biodegradability, biocompatibility, and multifunctional biological activities, have emerged as promising multifunctional tools for improving reproductive efficiency in farm animals by both direct and indirect mechanisms. Directly, they enhance reproductive processes by improving sperm quality during preservation, maintaining oocyte and embryo integrity during IVEP stages, and enhancing overall ART outcomes. CH-based drug delivery systems represent cost-effective, environmentally sustainable strategies for improving reproductive efficiency while controlling drug release and minimizing hormone use in the livestock industry. In addition, CH exhibits substantial antimicrobial and immunomodulatory potential for the management of uterine health, contributing to improved postpartum uterine recovery and reproductive performance. CH also positively influences gametogenesis, endocrine profile, placental efficiency, fetal development, offspring viability, and postnatal growth and productivity.

Indirectly, CH and CH-based materials improve reproductive potential by modulating gut health, rumen fermentation, nutrient utilization, metabolic efficiency, and oxidative balance, all of which are critical for fertility in high-producing animals under environmental and nutritional stress. Although the current evidence supports the use of CH and CH-based materials as a safe and sustainable strategy for modern livestock reproductive management, further large-scale, species-specific, and long-term investigations are required to optimize dosage, formulation, and mechanistic understanding. Incorporating CH-based materials into precision livestock management may provide a sustainable approach to boost reproductive performance, animal health, and productivity, even under challenging environmental conditions.

## Figures and Tables

**Table 4:** Applications of chitosan (CH) and chitosan-based materials for improving uterine health.

CH Form	Species	Case	Application Method	Optimal Dose/Outcomes	Proposed Mechanism of CH
CH-NPs [124]	Cattle (dairy cows)	Clinical/subclinical endometritis	In vitro, by the intrauterine fluid	0.5–2% CH-NPs added to bacterial isolates from endometrial fluid: ↓ *P. aeruginosa* and *Bacillus* sp. colony counts ↓ *S. aureus* colonies	CH-NPs act as an antibacterial biopolymer & non-toxic compound Interact with bacterial cell surfaces, altering membrane permeability & reducing growth Reduce colony count Do not impact molecular targets
CH microparticles [35,123,125,126,127]	Cattle (dairy cows)	Postpartum metritis	In vitro by intrauterine fluid & in vivo by intrauterine infusion	0.2% CH microparticles (8 gm/4 L uterine content), in vitro (added to metritic uterine fluid) or in vivo: Inhibit IUPEC in uterine fluid (In vitro) Eliminate IUPEC from the uterus by day 5 of treatment (In vivo) ↑ Clinical cure rates compared to ceftiofur ↓ Pathogenic families: *Fusobacteriaceae* & *Bacteroidaceae* by day 6 Shift the uterine microflora toward a healthy profile	CH microparticles exhibit antimicrobial activity at neutral uterine pH: Particles bind to bacterial OmpA and LPSs Disrupted membrane integrity causes cell death Display broad-spectrum antimicrobial activity without inducing resistance or cross-resistance over repeated exposure Reduce pathogenic taxa (e.g., *Fusobacterium* & *Bacteroides* spp.) Allow non-pathogenic flora to persist to rebalance the uterine microbiota Reduce postpartum bacterial load, reflected by higher uterine pH (preventive effect)
	Cattle (dairy cows)	Postpartum metritis prevention	Intrauterine infusion	0.2% CH microparticles 24 h after calving for 5 days: ↑ Uterine discharge pH ↓ Metritis incidence up to 7 DIM Safe & partially effective for metritis prevention	
	Cattle (dairy cows)	Clinical metritis	Intrauterine infusion	0.6% CH microparticles: ↓ Microbiome recovery toward a healthy state Did not act as an effective alternative to ceftiofur	The adverse effect due to increased intrauterine inflammation at the higher CH dose & reduced effectiveness against *Fusobacterium necrophorum* at 0.6%, despite in vitro inhibition
	Cattle (Primiparous dairy cows)	Clinical metritis in early lactation	Intrauterine infusion	24 gm CH microparticles in 40 mL sterile water on days 0, 2 & 4 following metritis diagnosis: ↓ Rumination & activity Not an effective treatment	The manuscript infers that CH’s immunostimulatory & pro-inflammatory properties may cause this impaired inflammatory response in the uterus, rather than providing a beneficial antimicrobial effect under the tested dose
	Cattle (dairy cows)	Clinical metritis	Intrauterine infusion	0.6% CH microparticles (24 gm/4 L uterine content) Not an effective treatment for metritis ↓ Cure risk compared to ceftiofur ↓ Milk yield, survival & fertility ↑ Uterine-related culling	Using CH microparticles at this dose seems likely to exacerbate uterine inflammation rather than provide clinically beneficial antimicrobial control
Raw CH solution [128,129,130]	Cattle (dairy cows)	Postpartum clinical endometritis	Intrauterine infusion	0.8% CH solution (50 mL) at week 3 postpartum in cows with endometritis: ↓ PMN% between week 3 and week 5 ↑ Uterine recovery	CH promotes wound healing by inducing PMN migration ↑ Local biological defense mechanisms ↑ Local immune responses Provides antimicrobial activity in the uterus ↑ Infection clearance & uterine recovery
	Cattle (dairy cows) (Anestrus)	-	Intrauterine infusion	Using CH solution before estrus/ovulation synchronization: ↑ Conception rate in anestrus cows after synchronization	CH exhibits local antimicrobial activity ↑ Uterine immune function, including PMN migration & ↑ biological defense Normalizes the uterine environment before synchronization, thereby improving conception rates
	Cattle (beef cows)	Postpartum anestrus	Intrauterine infusion	250 mg CH in 50 mL at 30 days after gestation: ↑ Estrus returns postpartum ↑ Reproductive efficiency	CH acts as an antibacterial agent ↑ Immune functions & uterine involution/recovery from postpartum inflammation, thereby shortening the interval to estrus return

↑: Increase, ↓: Decrease, CH: Chitosan, CH-NPs: Chitosan nanoparticles, DIM: Days in milk, IUPEC: Intrauterine pathogenic *E. coli*, LPSs: Lipopolysaccharides, OmpA: Outer membrane protein, PMN: Polymorphonuclear leukocyte.

**Table 5:** Applications of chitosan (CH) and chitosan-based materials as dietary supplementation directly impacted reproductive performance.

CH Form	Species	Outcomes	Proposed Mechanism of CH
Male			
Astaxanthin–CH-NPs [131]	Rabbit (bucks)	100–150 mg/kg diet as a feed additive ↑ Heat tolerance & physiological response & antioxidant status ↑ Semen volume, concentration, progressive motility, viability, integrity, seminal fructose & biochemical patterns Maintains testicular histology, active spermatogenesis & sperm ultrastructure ↑ Pregnancy rates in females mated to treated males	Astaxanthin–CH-NPs act as an antioxidant, anti-inflammatory & anti-apoptotic agent: ↑ Astaxanthin delivery, solubility & cellular uptake Scavenges ROS & RNS ↓ Lipid peroxidation (↓ MDA & H_2_O_2_) Binds to active sites of SOD, GPx & Nrf2, supporting modulation of antioxidant pathways ↑ Libido & fertility (↑ testosterone & ↓ reaction time)
Raw CH [132,133]	Goat (bucks)	2.5 gm CH/kg concentrate in normal or high-fat diet ↑ Ejaculate volume, progressive motility, viability, sperm concentration ↓ Abnormality, necrosis & DNA fragmentation ↑ Antioxidant defense ↑ Seminal plasma testosterone is higher	CH acts as an antioxidant, anti-inflammatory & nontoxic agent: ↑ GSH, CAT & GPx activity Scavenges free radicals & ROS ↓ MDA & lipid peroxidation Protects spermatogenesis & DNA integrity from a high-fat diet Modulates testicular function (↑ seminal testosterone, possibly via FSH & ABP binding) ↑ Nutrient transport/protection of the intestinal barrier ↓ Apoptosis & OS ↑ Steroidogenesis (testosterone & E_2_) Chelates toxins ↓ Cholesterol absorption ↑ Digestive/immunomodulatory effects for better spermatogenesis & semen quality
	Rabbit (bucks)	0.2–0.4 gm/kg CH as supplementation in basal pelleted ration: ↑ Testicular histology & spermatogenesis ↑ Basic & sexual behaviors ↑ Semen quality (volume, motility, concentration & intact acrosome) ↑ Reproductive efficiency	
Female			
Raw CH [134]	Rabbit (does)	0.2–0.4 gm/kg CH as supplementation in basal pelleted ration: ↑ Ovarian activity & development ↑ Receptivity rate & ↓ receptivity time ↑ Conception rate, ↓ kindling interval & ↑ parities	CH acts as an antioxidant, anti-inflammatory & nontoxic agent: ↑ Fermentation & nutrient utilization ↑ Immunoglobulins (IgA-IgG-IgM) ↑ Ovarian structure & activity Sustains steroidogenesis for better receptivity & conception
COSs (enzymolyzed CH) [42,135,136,137,138,139,140,141,142,143,144]	Swine (sows)	40 mg/kg COSs in corn–soybean basal diet during gestation (from estrus/mating to farrowing): ↑ Total born, viability & embryonic development ↑ Placental development & implantation ↑ Milk oligosaccharides & sialylation ↑ Piglet gut health/pathogen protection	↑ Embryonic survival & implantation via regulating endocrine & intrauterine environment ↑ Placental angiogenesis, nutrient transport & fetal growth (via GH & IGF-I upregulation) Alters milk oligosaccharides profile ↓ Adhesion of *E. coli* & toxins
	Swine (pregnant sows)	100 mg/kg COSs in corn–soybean basal diet early gestation (26-day post-mating to farrowing) ↑ Fetal survival rate & fetal development ↓ Stillborn/mummified ↑ Maternal immunity (↑ immunoglobulins (IgG/A/M)) ↑ Antioxidant defense (↑ TAC & ↓ MDA) ↑ Placental development & function	COSs act as an antioxidant, immunomodulatory, green antibiotic alternative that has anti-inflammatory and milk-mediated effects and is immune-enhancing ↑ Antioxidant defense by ↑ TAC & ↓ MDA Scavenges radicals by bioactive amino & hydroxyl groups ↑ IgG/A/M, IL-1 & TNF-α ↑ Placental angiogenic & growth factor by ↑ leptin & VEGFA ↑ Fetal development & survival genes Offering an optimal gestation environment reduces prenatal loss ↑ Resistance to gestation/lactation OS ↑ Colostrum IgM and trends in IgG & ↑ passive immunity ↑ Energy/nutrient supply, supporting piglet growth (during late gestation)
		100 mg/kg COSs in corn–soybean basal diet during late gestation (day 85 of gestation to farrowing) ↑ Average daily litter gain & piglet weaning weight/litter ↑ Antioxidant defense (↑ CAT, TAC, GPx & MDA) ↑ Immunity status: ↑ IL-10, IgA, IgM, IL-6, IgG ↑ Colostrum/milk composition	
		100 mg/kg COSs in corn–soybean basal diets of late gestation (from day 85 of gestation) and lactation: ↓ Stillborn, mummies/born dead ↓ Intrauterine growth restriction ↑ Placental function, nutrient transport & angiogenesis ↓ OS & inflammation	↑ Placental nutrient transport & angiogenesis (↑ VEGF-A, ↓ Ang-2) ↓ OS (↓ MDA, ↑ GSH/ATP, GSH/GSSG) ↓ Inflammation (↑ IL-10) In jejunum (piglet): ↑ Digestion, barrier integrity, anti-inflammation, antioxidant COS antioxidant, anti-inflammation, antibacterial effects are transmitted maternally via placenta/milk/microbiota to mitigate IUGR & OS damage
		100 mg/kg COSs in corn–soybean basal diet of gestation (from day 35 of gestation): ↑ Pathways associated with oocyte maturation & cell cycle regulation	COS supplementation Modulates ovarian function & metabolism for fecundity ↑ Pathways promote oocyte meiosis, maturation & cell cycle (P_4_-mediated, cyclin regulation) ↑ Embryo development, angiogenesis & survival markers
		100 mg/kg COSs in corn–soybean basal diet of gestation (from mating day to day 35 of gestation): ↑ The amniotic environment improves fetal survival/growth ↑ Antioxidant, immune & metabolic status ↑ Fetal survival rate & fetal development	↑ Antioxidant defense (SOD, CAT & TAC, scavenging ROS) ↑ Anti-inflammation/immune (IL-10/IgG/IgM protects feto-placental) ↓ OS & embryonic loss
		30 mg/kg COSs in corn–soybean basal diet of late gestation (from day 85 of gestation to parturition): ↑ Placental function & placental amino acid transport for fetal health ↑ Antioxidant defense & fetal growth	↑ Plasma/placental antioxidants (SOD/CAT) Scavenge ROS ↓ Inflammation (IL-6/8/1β) ↑ Amino acid transporters ↑ VEGF-A mRNA for nutrient transfer, placental function & fetal growth
		100 mg/kg COSs in the basal diet during gestation (from day 90 of gestation to weaning): ↑ Reproductive performance ↓ Weaning-to-estrus interval ↑ Milk composition ↑ Piglets’ gut growth, immunity, microbiota, & short-chain fatty acids	COSs are a water-soluble, biocompatible, gut-absorbed agent ↑ Epithelial differentiation, metabolic activity Modulates gut microbiota ↑ Milk bioactive components, nutrients & TGF-β, enhancing passive immune transfer ↑ Placental prior benefits extend to long-term gut programming and development
		30 mg/kg COSs in corn–soybean basal diets of late gestation & lactation (from day 85 of gestation to day 14 of lactation): ↑ Piglet small intestine development ↑ Antioxidant capacity	Scavenges ROS (H_2_O_2_, hydroxyl radicals & singlet O_2_) ↑ Plasma GPx helps to protect piglets against OS at birth ↑ Intestinal antioxidant genes (SOD, GPx & CAT) ↑ Growth & oxidative status may also modulate subsequent liver function and development
		30 mg/kg COSs in corn–soybean basal diets of late gestation & lactation (from day 85 of gestation to day 14 of lactation): ↑ Average weaning weight & growth rate of piglets ↑ Milk composition ↑ Hepatic gluconeogenesis & ↓ hypoglycemia in piglets ↑ Hepatic free fatty acids ↑ Piglet small intestine development ↑ Antioxidant capacity	COSs modulate energy metabolism ↑ Sow milk supply of glucogenic amino acids, providing more substrates for piglet hepatic gluconeogenesis ↑ Hepatic free fatty acids directly stimulate transcription and activity of gluconeogenic enzymes ↑ Glycogenolysis and gluconeogenesis, thereby raising plasma glucose and ↓ reliance on limited glycogen stores ↑ Energy availability supports the growth of suckling piglets
	Swine (pregnant gilts)	0.12 g/gilt/d COSs during gestation and 0.24 g/gilt/d during lactation: ↑ Late gestation BW gain & 2nd pregnancy rate ↑ Milk & suckling bout ↑ Offspring growth & immunity	COSs act as an immunostimulant, antioxidant & anti-inflammatory agent ↑ Mammary cell proliferation & milk synthesis Improves passive immunity transfer (↑ colostrum Ig) ↑ Gut mucosa protection (↑ IgA blocks pathogens) ↑ Growth factors (GH/IGF-1) ↑ Suckling intensity (↑ oxytocin & milk let-down)

↑: Increase, ↓: Decrease, ABP: Androgen-binding protein, Ang-2: Angiopoietin-2, ATP: Adenosine triphosphate, BW: Body weight, CAT: Catalase, CH: Chitosan, CH-NPs: Chitosan nanoparticles, COS: Chitosan oligosaccharide, *E. coli*: *Escherichia coli*, E_2_: Estradiol, FSH: Follicle-stimulating hormone, GH: Growth hormone, GPx: Glutathione peroxidase, GSH: Reduced glutathione, GSSG: Oxidized glutathione, H_2_O_2_: Hydrogen peroxidase, IGF-1: Insulin-like growth factor 1, MDA: Malondialdehyde, mRNA: Messenger RNA, Nrf2: Nuclear factor erythroid 2-related factor 2, O_2_: Oxygen, OS: Oxidative stress, P_4_: Progesterone, RNS: Reactive nitrogen species, ROS: Reactive oxygen species, SOD: Superoxide dismutase, TAC: Total antioxidant capacity, TGF-β: Transforming growth factor beta, TNF-α: Tumur necrosis factor alpha, VEGF-A: Vascular endothelial growth factor A.

## Data Availability

No new data were created or analyzed in this study.

## Abbreviations

The following abbreviations are used in this manuscript:

AI	Artificial insemination
ARTs	Assisted reproductive technologies
Bax	Bcl-2-associated X protein
Bcl-2	B-cell lymphoma 2 (anti-apoptotic)
BMP15	Bone morphogenetic protein 15
brIFN τ	Bovine recombinant interferon tau
BW	Body weight
CAT	Catalase
CH	Chitosan
CH-NPs	Chitosan nanoparticles
CIDR	Controlled internal drug release
CL	Corpus luteum
COS	Chitosan oligosaccharide
DD	Degree of deacetylation
DM	Dry matter
DMI	Dry matter intake
DPPH	Diphenyl picrylhydrazyl
*E. coli*	*Escherichia coli*
E_2_	Estradiol
FGFR2	Fibroblast growth factor receptor 2
FSH	Follicle-stimulating hormone
GDF9	Growth differentiation factor 9
GnRH	Gonadotropin-releasing hormone
GPx	Glutathione peroxidase
GSH	Reduced glutathione
hCG	Human chorionic gonadotropin
ICSI	Intracytoplasmic sperm injection
Ig(A/M/G)	Immunoglobulins
IGF-1	Insulin-like growth factor 1
IL-(6/8/10/1β)	Interleukins
iNOS	Induced nitric oxide synthase
IVC	In vitro culture
IVEP	In vitro embryo production
IVM	In vitro maturation
LH	Luteinizing hormone
LPSs	Lipopolysaccharides
MDA	Malondialdehyde
MW	Molecular weight
NF-κB	Nuclear factor kappa-B
NGF	Nerve growth factor
NO	Nitric oxide
NPs	Nanoparticles
Nrf2	Nuclear factor erythroid 2-related factor 2
OmpA	Outer membrane protein
OS	Oxidative stress
OVS	Ovsynch
P_4_	Progesterone
PCL	polycaprolactone
PEG	Polyethylene glycol
PGF_2α_	Prostaglandin
PMN	Polymorphonuclear leukocyte
PMSG	Pregnant mare gonadotropin
PRID	Progesterone-releasing intravaginal device
ROS	Reactive oxygen species
rβ-NGF	Recombinant β-nerve growth factor
SOD1	Superoxide dismutase 1
SOD	Superoxide dismutase
STAT3	Signal transducer and activator of transcription 3
TAC	Total antioxidant capacity
TGF-β	Transforming growth factor beta
THI	Temperature–humidity index
TNF-α	Tumor necrosis factor alpha
TPP	Sodium tripolyphosphate
VEGF-A	Vascular endothelial growth factor A
VFAs	Volatile fatty acids
